# The human oral – nasopharynx microbiome as a risk screening tool for nasopharyngeal carcinoma

**DOI:** 10.3389/fcimb.2022.1013920

**Published:** 2022-11-30

**Authors:** Yu Hao, Zhi Zeng, Xian Peng, Ping Ai, Qi Han, Biao Ren, Mingyun Li, Haohao Wang, Xinxuan Zhou, Xuedong Zhou, Yue Ma, Lei Cheng

**Affiliations:** ^1^ State Key Laboratory of Oral Diseases & West China Hospital of Stomatology & National Clinical Research Center for Oral Diseases, Sichuan University, Chengdu, China; ^2^ Department of Operative Dentistry and Endodontics, West China School of Stomatology, Sichuan University, Chengdu, China; ^3^ Head & Neck Oncology Ward, Cancer Center, West China Hospital, Sichuan University, Chengdu, China; ^4^ Division of Radiotherapy, Cancer Center, West China Hospital, Sichuan University, Chengdu, China; ^5^ Department of Oral Pathology, West China School of Stomatology, Sichuan University, Chengdu, China; ^6^ West China School of Public Health and West China Fourth Hospital, Sichuan University, Chengdu, China

**Keywords:** nasopharyngeal carcinoma, screening, microbiome, 16S rDNA sequencing, random forest

## Abstract

Nasopharyngeal carcinoma (NPC) is a common head and neck cancer with a poor prognosis. There is an urgent need to develop a simple and convenient screening tool for early detection and risk screening of NPC. 139 microbial samples were collected from 40 healthy people and 39 patients with nasopharyngeal biopsy. A total of 40 and 39 oral, eight and 27 nasal cavity, nine and 16 nasopharyngeal microbial samples were collected from the two sets of individuals. A risk screening tool for NPC was established by 16S rDNA sequencing and random forest. Patients with nasopharyngeal biopsy had significantly lower nasal cavity and nasopharynx microbial diversities than healthy people. The beta diversity of the oral microbiome was significantly different between the two groups. The NPC screening tools based on nasopharyngeal and oral microbiomes have 88% and 77.2% accuracies, respectively. The nasopharyngeal biopsy patients had significantly higher *Granulicatella* abundance in their oral cavity and lower *Pseudomonas* and *Acinetobacter* in the nasopharynx than healthy people. This study established microbiome-based non-invasive, simple, no radiation, and low-cost NPC screening tools. Individuals at a high risk of NPC should be advised to seek further examination, which might improve the early detection of NPC and save public health costs.

## Introduction

Nasopharyngeal carcinoma (NPC) is an aggressive malignant tumor of the head and neck mucosal epithelium. In 2018, 129,100 new NPC cases were reported worldwide (Ferlay et al., 2019), with over 70% of the cases in Southeast Asia (Chen et al., 2019). Severe NPC symptoms include headache, nasal bleeding, nasal congestion, hearing loss, and neck mass. Given that these symptoms are unspecific, NPC misdiagnosis is a common phenomenon (Kamran et al., 2015). Consequently, 80% of clinically confirmed NPC patients have locally advanced or distant metastases, all with poor prognoses (Chan et al., 2017; Lee H. M. et al., 2019). The 5-year survival rate of stage I and IV NPC are 90% and 60%, respectively (Li et al., 2014). Early diagnosis and treatment are vital for a good NPC prognosis, underscoring the need for early screening of NPC (Lee A.W.M. et al., 2019).

Clinical NPC diagnosis involves biopsy and imaging examinations, including endoscopy, Magnetic Resonance Imaging (MRI), Computed Tomography (CT), and ultrasound. Nasopharyngeal endoscopy examines the lesion range, and a biopsy is taken using forceps. MRI, CT, and ultrasound show nasopharyngeal lesions, invasion, and metastasis (King et al., 2011; Gao et al., 2014). However, these methods are expensive, require experienced personnel and expose patients to ion radiation. Presently, conventional NPC diagnosis methods are inaccurate for early screening. Thus, there is an urgent need for non-invasive, simple, and low-cost NPC screening tools.

Numerous factors, including genetics, dietary habits of preserved food, and Epstein-Barr virus (EBV) infection, have been implicated in the development of NPC (Tsao et al., 2017; Lee H. M. et al., 2019). Persistent nasopharyngeal mucosal inflammation and certain medications increase the risk of NPC (Xiao et al., 2018; Zhang et al., 2019). Recently, there has been increasing interest in the association between the microbiome and NPC. A retrospective study showed that poor oral hygiene, including infrequent brushing and frequent dental caries, increases the risk of NPC (Turkoz, 2011; Liu et al., 2016). Another study showed that the relative abundance of *Firmicutes* and *Streptococcus* was lower in the saliva of NPC patients than in healthy controls (Xu et al., 2014). The cytolethal distending toxin of *Aggregatibacter actinomycetemcomitans*, oral pathogenic bacteria, reactivates EBV and triggers DNA destruction. Thus, co-infection with bacteria and viruses can cause EBV-induced epithelial malignancy, including NPC (Frisan et al., 2018). A case-control study of 499 NPC patients and 495 healthy controls found that oral microbiome richness was significantly low in NPC patients (*P* < 0.001). The study revealed *Granulicatella adiacens*, a possible disease-related species (Debelius et al., 2020). These case studies suggest an aberrant oral microbial composition in NPC patients. However, the NPC screening tools of the oral microbiome are lacking.

This study analyzed the microbiome of the oral and nasal cavities, and the nasopharynx from people with high NPC risk, requiring a nasopharyngeal biopsy and healthy controls. The NPC risk screening tool was established based on the16S rDNA sequencing and the random forest.

## Materials and methods

### Ethics statement and subjects

The experimental protocol of this study was approved by the ethics committee approved the (Approval number: WCHSIRB-D-2018-101). Previous studies (Xiao et al., 2018; Zhang et al., 2019) revealed that persistent nasopharyngeal mucosal inflammation increases the risk of NPC development, and a nasopharyngeal biopsy of such patients should be further analyzed for a definitive diagnosis. The inclusion criteria included: patients needing a nasopharyngeal biopsy and healthy people confirmed by nasopharyngeal fiberscope, aged 20-70 years old, and provided informed consent before sample collection.

The exclusion criteria were: antibiotics use within three months; a history of dental treatment within six months; less than 20 natural teeth; over six DMFT (decayed, missing, or filled teeth); severe oral disease (periodontal diseases, oral fungal infection, and oral cancer); pregnant or nursing women; a family history of cancer; and having smoked over 100 cigarettes in a lifetime (Yu et al., 2017).

### Sample collection

As in a previous study (Hao et al., 2021), the participants included 40 healthy people and 39 patients with nasopharyngeal biopsy who were advised not to eat, drink, or chew gum 30 min before sampling. A 5 mL of saliva from each study participant was collected in a sterile centrifuge tube. After centrifugation (2600×g, 10min), the saliva was stored in a 2 mL cryogenic tube and immediately frozen at −80°C.

Nasopharyngeal microbiome were collected by nasopharyngeal swabs from nine healthy individuals and 17 patients with nasopharyngeal biopsies. The sterile nasopharyngeal swab was dipped in sterile saline and wrapped in a sterile infusion tube, passed through the nasal cavity into the nasopharynx. While in the nasopharynx, the swab was extended and rotated through 360° to dip in nasopharyngeal secretions. The swab was then withdrawn into the infusion tube, which was removed altogether. The swab tip was cut off with sterile scissors, stored in a cryogenic tube, and immediately frozen at −80°C. The microbiome of the nasal cavity was collected from eight healthy people and 27 patients with nasopharyngeal biopsy. Two swabs dipped in sterile saline were used to collect the microbiome in the left and right noses. The swabs were rotated and wiped the anterior nostril mucosa. The swab tip was cut off with sterile scissors, stored in a cryogenic tube, and immediately frozen at −80°C (Luna et al., 2018).

### Illumina MiSeq sequencing

The collected microbiome samples above were used for DNA extraction, amplification, and 16S rDNA sequencing (Wang et al., 2016; Yin et al., 2016). Microbial DNA was extracted using the E.Z.N.A.^®^ soil DNA Kit (Omega Bio-Tek, USA) following the manufacturer’s protocol. The V4-V5 target regions of the bacterial 16S rRNA gene were amplified *via* polymerase chain reaction (PCR) with barcoded primers 515 F (5′-GTGCCAGCMGCCGCGG-3′) and 907R (5′−CCGTCAATTCMTTTRAGTTT-3′) (Wu et al., 2020).

Majorbio Bio-Pharm Technology Co., Ltd. (Shanghai, China) purified and pooled amplicons in equimolar amounts and sequenced paired-end (2 x 300bp) on the Illumina MiSeq platform (Illumina, CA, USA) following standard protocols. Raw reads were deposited to the National Center for Biotechnology Information(NCBI) Sequence Read Archive(SRA) database (Accession Number: PRJNA722880).

### Processing of sequencing data

Majorbio Bio-pharm Technology Co., Ltd. quality-filtered raw FASTQ files using Trimmomatic software and merged files using the FLASH software following the standard criteria. Operational taxonomic units(OTUs) were clustered with a 97% similarity cutoff using UPARSE Version 7.1 (http://drive5.com/uparse/) with a novel ‘greedy’ algorithm that simultaneously performs chimera filtering and OTU clustering. The taxonomy of each 16S rRNA gene sequence was analyzed using the Ribosomal Database Project(RPD) Classifier algorithm (http://rdp.cme.msu.edu/) against the Silva database using a 70% confidence threshold (Cole et al., 2005; Dewhirst et al., 2010) (**Table S4**, **Figure S3**).

### Statistical analysis

The states of nasopharynx were divided into healthy or tissue need biopsy according to the nasopharyngeal fiberscope. Then the nasopharyngeal biopsy group was further divided into cancer and inflammation groups based on pathological analysis (**Table S3**). Thus, the study participants were classified into healthy, inflammation, and cancer groups according to the states of the nasopharynx. The healthy and inflammation groups were integrated into non-cancer group, while the inflammation and cancer groups were integrated into the nasopharyngeal biopsy group.

The microbiome composition, alpha and beta diversity based on the genus level was established using the mother software (version.1.30.2.). Shannon indices were tested using the Wilcoxon rank-sum test, and the Principal Coordinates Analysis(PCoA) was performed based on the Bray_curtis distance methods. The differences among groups were determined using Analysis of Similarity (ANOSIM). Differences with *P*<0.05 were considered statistically significant. The R>0 was considered that there is a significant difference between the two sampling units.

Random Forest is a robust machine learning algorithm by combining the output of multiple decision trees to obtain a single outcome that could handle both classification and regression problems (Breiman, 2001). The random forest classifier and screened model establishment based on the high dimensional 16S rDNA sequencing data. One to three nasal and nasopharynx oral samples were used to establish the screening model considering the microbial community diversity from the different sample sources. The error rates of the training and verification sets were calculated. The optimal *n_tree_* value for establishing the random forest models with low and stable error rates were determined using multiple iterations. Repeated cross-validations with 80% training and 20% testing sets evaluated the model external accuracy. The weight, known as variable importance, was originally defined in the random forest using a measure involving surrogate variables, and it was calculated by randomly permuting a variable (Ishwaran, 2007). The weights of characteristic variables were calculated to correlate genus and outcomes according to the established optimal models.

## Results

### Characteristics and exploratory analysis of microbiome

The characteristics analysis was performed among the three microbiome habitats (oral, nasal and nasopharyngeal). The Shannon index of alpha diversity (**Figure S1A**) showed that the oral microbiome was the most diverse (*P*<0.001). PCoA analysis (**Figure S1B**) showed that the beta diversity of the oral, nasal, and nasopharyngeal microbiome was significantly different (*P*<0.05, R=0.9627). The Venn graph (**Figure S1C**) of species composition illustrated 599 common OTUs in the three groups, accounting for 25.72-37.94% of the total microbiome. The microbial community composition in the nasal cavity and nasopharynx was similar between the two groups. However, the oral microbial composition was different between the two groups (**Figure S1D**).

**Figure 1:**
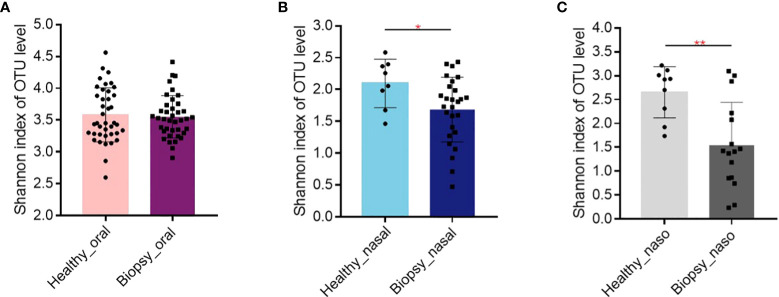
Alpha diversity of Shannon index. **(A)** The Shannon index of oral microbiome between patients with nasopharyngeal biopsy and healthy counterparts. **(B)** The Shannon index of nasal microbiome between patients with nasopharyngeal biopsy and healthy counterparts. **(C)** The Shannon index of nasopharyngeal microbiome between patients with nasopharyngeal biopsy and healthy counterparts. Oral represents oral microbiome, nasal represents nasal microbiome, naso represents nasopharyngeal microbiome. "*" represented P<0.05, "**" represented P<0.01.

The **Figure 1** shows the alpha diversity between healthy and biopsy groups. The oral microbiome of patients with nasopharyngeal biopsy and healthy counterparts were not significantly different (**Figure 1A**). As for nasal and nasopharyngeal microbiome (**Figures 1B, C**), the Shannon indices of patients with nasopharyngeal biopsy were significantly lower than healthy counterparts (*P*<0.05, *P*<0.01).

**Figure 2:**
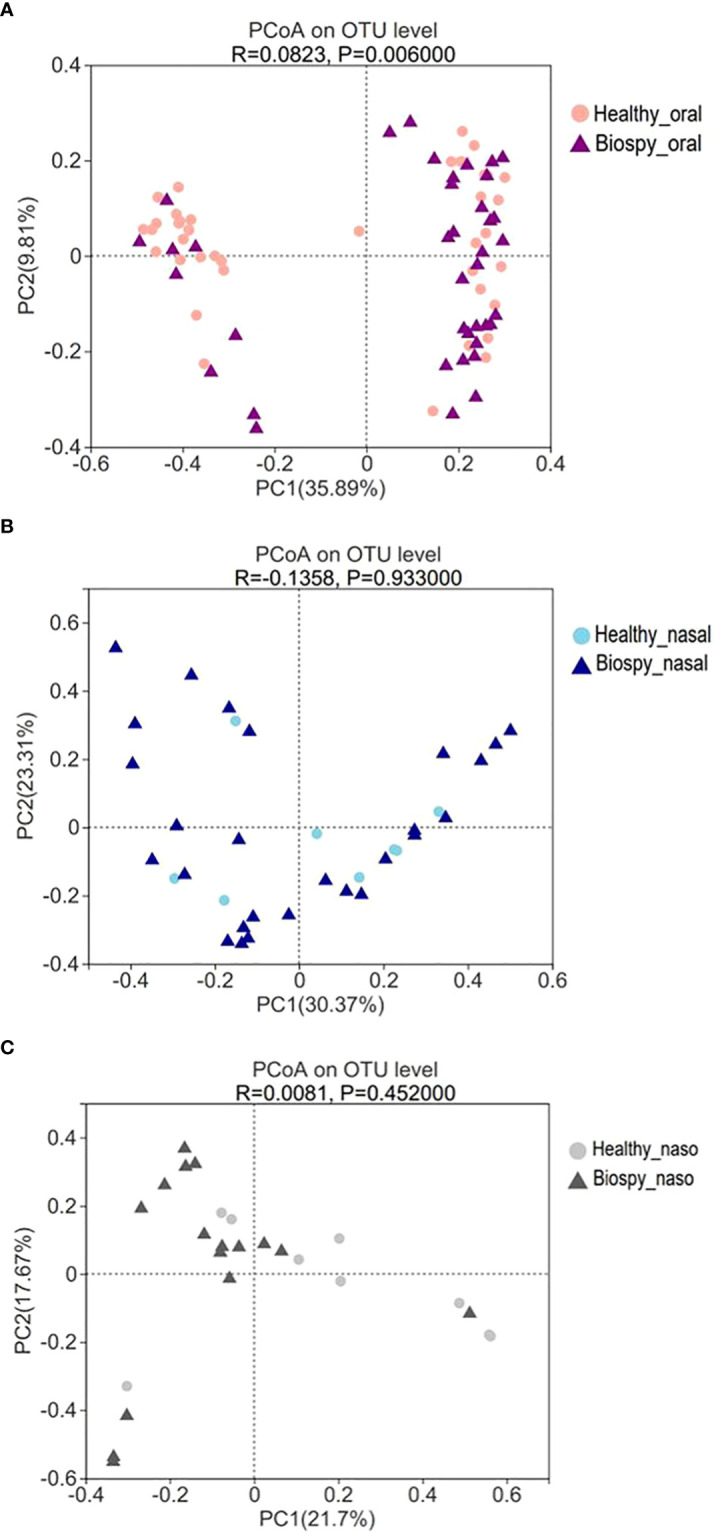
Beta diversity of PCoA analysis. **(A)** The PCoA analysis of oral microbiome between patients with nasopharyngeal biopsy and healthy counterparts. **(B)** The PCoA analysis of nasal microbiome between patients with nasopharyngeal biopsy and healthy counterparts. **(C)** The PCoA analysis of nasopharyngeal microbiome between patients with nasopharyngeal biopsy and healthy counterparts. Oral represents oral microbiome, nasal represents nasal microbiome, naso represents nasopharyngeal microbiome.

The PCoA based on Bray_curtis distance revealed that there was a significant difference in the beta diversity of oral microbiome between nasopharyngeal biopsy patients and healthy counterparts (R>0; *P*<0.05) (**Figure 2A**). However, there was no significant difference in the composition of the nasal cavity and nasopharyngeal microbiome between nasopharyngeal biopsy patients and healthy counterparts (**Figures 2B, C**).

The similarity of microbial composition among groups was determined using ANOSIM (**Table 1**). The oral microbiome showed a significant difference between the healthy and the nasopharyngeal biopsy groups (R>0; *P*<0.05). The diversity of the nasopharyngeal microbiome was significantly different of the healthy vs. cancer, and healthy vs. inflammation groups (R>0; *P*<0.05). However, the microbial composition in the oral, nasal cavity and nasopharynx was not significantly different between the inflammation and the cancer groups (*P*>0.05).

**Figure 3:**
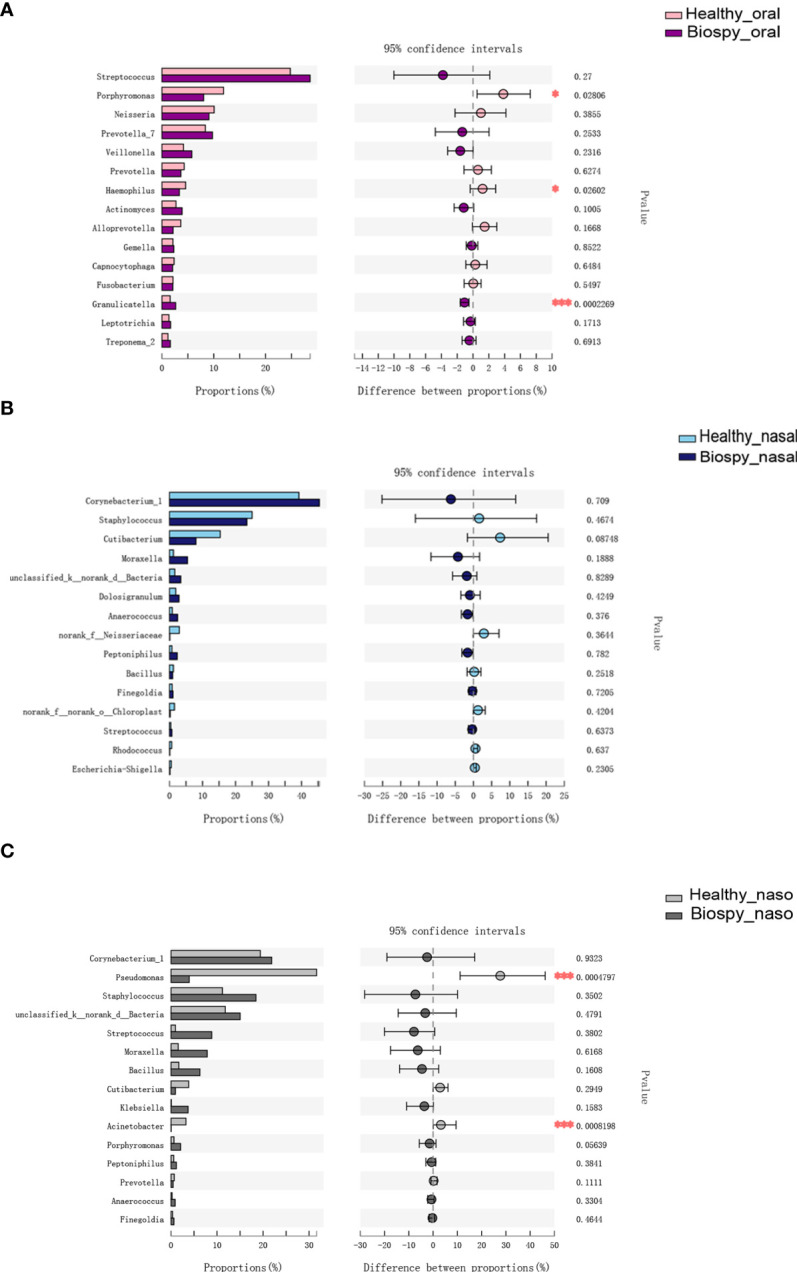
Genus level analysis of species differences. **(A)** Differences in the dominant species composition of oral microbiome between patients with nasopharyngeal biopsy and healthy counterparts. **(B)** Differences in the dominant species composition of nasal cavity microbiome between patients with nasopharyngeal biopsy and healthy counterparts. **(C)** Differences in the dominant species composition of nasopharyngeal microbiome between patients with nasopharyngeal biopsy and healthy counterparts. Oral represents oral microbiome, nasal represents nasal microbiome, naso represents nasopharyngeal microbiome. "*" represented P<0.05, "***" represented P<0.001.

**Figure 3A** shows the species differences in the oral microbiome on genus level. The relative abundance of *Granulicatella* genus was higher in the patients with nasopharyngeal biopsy than in healthy group (*P* < 0.001), but the *Porphyromonas* and *Haemophilus* were relatively less abundant (*P* < 0.05). There was no significant difference in the dominant microbial species in the nasal cavity between nasopharyngeal biopsy group and healthy counterparts (**Figure 3B**). Considering the nasopharynx, the relative abundances of *Pseudomonas* and *Acinetobacter* in the healthy counterparts were significantly higher than in patients with nasopharyngeal biopsy (*P* < 0.001, **Figure 3C**).

### NPC risk screening model based on oral or nasopharyngeal microbiome

The NPC risk screening models were established *via* random forests using the microbial sequencing data. The accuracy of the models are shown in **Table 2**. The accuracy of models based on different microbial combinations were approximately 80%. The accuracy of the risk screening model based on the microbiome from one habitat (the oral microbiome) was 77.22%. The Area Under Curve(AUC), sensitivity, and specificity of the model were 80.96%, 0.7692, and 0.775, respectively. For the risk screening model based on the nasopharyngeal microbiome, the accuracy was 88%. The AUC, sensitivity, and specificity of the model were 83.33%, 0.875, and 0.8889, respectively.

The external performance of the model was evaluated using repeated cross-validations. For each model, 80% random samples were used for model training and 20% samples for validating the external test sets. The average external accuracy of all random forests provided an estimate of the model applied in external populations. The average accuracy of the model was stable in a 5000 times validation. Based on the oral microbiome, the average accuracy rate of the screening model was 75.08%, and the median accuracy rate was 75%. The nasopharyngeal microbiome had an 85.22% average accuracy rate of the screening model and an 80% median accuracy rate.

The weights of characteristic variables were calculated following the above-built models. Considering the oral microbial model, the most important genera in weight value were *Graulicatella*, *Prevotella*, *Rhodococcus*, and *Haemophilus* (**Table S1**). *Granulicatella* ranked first and fourth, suggesting that this genus is a key biomarker of the oral microbiome. For the nasopharyngeal microbial model, *Candidatus solibacter*, *Pseudomonas*, *Bradyrhizobium*, and *Reyranella* were the most important genera, ranked by the front of weight value (**Table S2**). Genus *Pseudomonas* ranked the second, third, and seventh, suggesting it is a key biomarker of the nasopharyngeal microbiome.

## Discussion

Many recent studies revealed the close relationship between the microbiome and neoplasm (Flemer et al., 2018; Yang et al., 2018; Li et al., 2020). In some studies, the specific microbiome composition was used as a biomarker to evaluate the risk of cancers and establishing diagnostic models (Lim et al., 2018; Ren et al., 2018; Sze and Schloss, 2018; Zhou et al., 2021). The microbiome capability to serve as disease biomarkers varied with the specificity of the microbial composition at the disease and anatomical site (Nejman et al., 2020). This study established for the first time the NPC risk screening models using the oral and nasopharyngeal microbiomes. The accuracy rates of nasopharyngeal and oral microbiomes were 88% and 77.2%, respectively. This model can preliminarily screen the NPC high-risk group through a non-invasive microbial sample detection. For the high-risk group with detected NPC, invasive nasopharyngeal fiberscope and biopsy can be applied for further examination, a conducive strategy for early NPC detection.

The 16S rDNA sequencing showed that the alpha diversity of the oral microbiome displayed no significant difference between healthy and biopsy samples (**Figure 1A**), whereas the beta diversity was significantly different (**Figure 2A**). The Shannon index of oral microbiome was differed from that of previous study, which might due to different clustering methods and grouping (Debelius et al., 2020). The different beta-diversity suggested that the development of NPC shaped different microbiome in the oral cavity. Besides, there was no significant difference in the oral, nasal cavity, and nasopharynx microbial composition betwwen the inflammation and cancer groups (**Table 1**, *P*>0.05). The dominant species of the inflammation group were more similar to cancer than the healthy group (**Figure S2**). Studies revealed that persistent nasopharyngeal mucosal inflammation increases the risk of NPC (Xiao et al., 2018; Zhang et al., 2019). Inflammation produces reactive oxygen and nitrogen, which damage DNA and cause stem cell mutations, damage biological macromolecules, and induce cell dysfunction, promoting NPC occurrence and development (Murata, 2018). Thus, the inflammation group was a likely NPC high-risk group that needed further examination.

**Table 1:** Analysis of similarity among groups.

Microbiome	Groups	R value	P value
Oral	Hea vs. Bio	0.0823	0.006
	Can vs. non-Can	-0.0168	0.732
	Hea vs. Can	0.0015	0.405
	Hea vs. Inf	-0.0083	0.481
	Inf vs. Can	-0.0456	0.7
Nasal Cavity	Hea vs. Bio	-0.1358	0.933
	Can vs. non-Can	0.0767	0.066
	Hea vs. Can	-0.0612	0.705
	Hea vs. Inf	-0.077	0.839
	Inf vs. Can	0.0369	0.211
Nasopharynx	Hea vs. Bio	0.0081	0.452
	Can vs. non-Can	0.1041	0.131
	Hea vs. Can	0.1877	0.029
	Hea vs. Inf	0.2832	0.003
	Inf vs. Can	0.0611	0.239

Hea represented the healthy group, Bio represented nasopharyngeal biopsy group, Can represented the cancer group, Non-can represented the non-cancer group, Inf represented the inflammation group.

Traditional analysis methods for establishing the risk screening tools based on the microbial community suffer numerous limitations and challenges for the multi-dimensionally complex microbial sequencing data (Quince et al., 2009; Hu et al., 2013). Random forests are effective tool in prediction which can effectively analyze large and noisy datasets with small samples and build classification models (Caruana and Niculescu-Mizil, 2006; Teng et al., 2015; Zhu et al., 2017). Random forests models do not overfit because of the law of large numbers. Following the right kind of randomness makes them accurate classifiers. (Breiman, 2001; Doupe et al., 2019). The performances of random forests vary among diseases (Teng et al., 2015; Ren et al., 2018; Zhou et al., 2021). This study established the NPC risk screening tools using a non-invasive approach based on microbial sample detection for the first time.

Risk screening tools based on different sample sources showed different performances (**Table 2**). The oral or nasopharyngeal microbiome showed good screening capability with balanced sensitivity and specificity based on the microbiome from one habitat. The oral microbiome tool identified approximately 77% of the NPC high-risk population that needed further examination. The nasopharyngeal microbiome tool identified approximately 88% of the NPC high-risk population requiring further examination. Although the specificity of the EBV serology test was 97.12%, which is very high compared with the commonly used screening tool, its positive predictive value was only 4.41%, even in the high prevalence areas (Li et al., 2018). The positive predictive value of the microbiome-based screening tools of the oral and nasopharyngeal microbiome was 76.9% and 93.3% respectively. The highest sensitivity with a 0% false-negative rate was for the nasal microbiome, conducive for initial NPC screening to avoid missed diagnosis. Similarly, screening tools based on the combination of nasal and other habitats showed higher sensitivities. Meanwhile, higher specificities were registered, and more than half of patients were misdiagnosed. A previous study (Zhou et al., 2021) revealed that more samples improve the performance of the screening tools, especially the nasopharyngeal samples.

**Table 2:** Accuracy rates of NPC risk screening models.

Microbial Samples	Accuracy rate	AUC	Sensitivity	Specificity
139 samples (oral+nasal+nasopharyngeal)	79.86%	0.85	0.8537	0.7193
114 samples (oral+nasal)	78.95%	0.82	0.8636	0.6875
104 samples (oral+nasopharyngeal)	78.85%	0.85	0.7818	0.7959
60 samples (nasal+nasopharyngeal)	83.33%	0.83	0.9535	0.5294
79 samples (oral)	77.22%	0.81	0.7692	0.775
35 samples (nasal)	82.86%	0.66	1	0.25
25 samples (nasopharyngeal)	88%	0.83	0.875	0.8889

Following the weight values order of characteristic variables, the id ranked first, suggesting that the microbiome composition varied among individuals (Zhu et al., 2020). Besides, the weights of characteristic variables provided potential NPC biomarkers. In the oral microbial model, the genus *Granulicatella* ranked first and fourth (**Table S1**), showing higher relative abundance (*P* < 0.001) in the oral habitat of patients with nasopharyngeal biopsy, consistent with Debelius et al. findings, which suggested that the oral *Granulicatella adiacens* variant from an individual was significantly associated with microbial community structure and may be a way by which NPC status shaped the oral microbiome. (Debelius et al., 2020). The oral microbiome produced carcinogenic metabolites such as nitrosamines. *Granulicatella adiacens* mentions genes related to nitrate and nitrite reduction (Hyde et al., 2014). Carcinogens such as nitrosamines, produced by chemical reactions of nitrates and nitrites, can initiate and stimulate cancer progression through various mechanism including inflammation, DNA damage, oxidative stress, cytotoxicity, and subsequent regenerative proliferation *via* apoptosis (Song et al., 2015; Fishbein et al., 2021).

In the nasopharyngeal microbial model, genus *Pseudomonas* ranked the second, third and seventh among the weight values order (**Table S2**). The relative abundance of *Pseudomonas* and *Acinetobacter* significantly decreased in the patients with nasopharyngeal biopsy (*P* < 0.001, **Figure 3C**). Similar microbial changes have been observed in the gut microbiome of patients with colorectal cancer (Yang et al., 2019). *Acinetobacter* and *Pseudomonas* are opportunistic pathogens associated with nosocomial infection (Mwangi et al., 2019). However, the abundance of *Pseudomonas* and *Acinetobacter* was significantly low in the NPC high-risk group. It was illustrated that *Acinetobacter* species play a significant immunomodulatory and anti-inflammatory role in the skin microbiome (Fyhrquist et al., 2014). The high cytotoxic potential of *Pseudomonas* exotoxin A (PE) can generate anti-tumor immunotoxins for targeting tumor-associated antigens (Wolf and Elsasser-Beile, 2009). The *Pseudomonas aeruginosa* exopolysaccharides demonstrated anti-tumor activity against HT-29 colorectal cancer cells (Tahmourespour et al., 2020), while *Pseudomonas aeruginosa* L-asparaginase showed strong anti-tumor activity against HeLa cells (Fatima et al., 2019). Besides, *Pseudomonas fluorescens* lectin (PFL) inhibited neovascularization in a dose-dependent manner and downregulated the integrin and epidermal growth factor receptor, inhibiting *in vitro* and *in vivo* tumor growth (Sato et al., 2016; Sato et al., 2019). Altogether, the abovementioned potential biomarkers provide the preliminary basis for further studying the NPC-microbiome relationship.

The nasopharynx has a higher proportion of potentially pathogenic microorganisms than the nasal cavity (De Boeck et al., 2017). The nasopharyngeal microbiome was *in situ* of NPC, from which the model accuracy reached 88%. Although the nasopharyngeal microbiome sampling causes discomfort, the recent popularity of COVID-19 detection using the nasopharyngeal swab (Seo et al., 2020) has improved sampling acceptability. Thus, the nasopharyngeal microbiome can be used for NPC risk screening.

Saliva can be collected non-invasively and easily. The inorganic components, antioxidants, hormones, antibodies, and antigens in the saliva are biomarkers for diagnosing various oral and systemic diseases (Ngamchuea et al., 2017; Gerner et al., 2019). Specific microbiome changes in saliva are potential diagnostic biomarkers for head and neck cancers, including oral and hypopharyngeal cancers (Lim et al., 2018; Panda et al., 2020). Additionally, the oral microbiome predicts early childhood caries with 81% accuracy (Teng et al., 2015) and also predicts the oral mucositis progression after NPC radiotherapy (Zhu et al., 2017). In this study, the oral microbiome accuracy rate for NPC risk screening reached 77.2%. There is need to establish a non-invasive and convenient oral microbial sampling model to screen various diseases in a large population simultaneously. Therefore, this model has a high prospect of clinical application and could be applied in other regions with high disease incidence.

## Conclusion

This study established a non-invasive and low-cost NPC risk screening model based on the oral and nasopharyngeal microbiome. The present study demonstrated characteristic microbial diversities varied from different nasopharynx status in the oral and nasopharyngeal microbiome. Future studies with a larger cohort from the different regions are needed to validate and promote the efficacy of oral microbiome-based NPC screening. Generally, we developed an accurate model with specific microbial markers for early screening of the risk of NPC, which guides further confirmatory examination to achieve early diagnosis and early therapy for NPC.

## Data availability statement

The datasets presented in this study can be found in online repositories. The names of the repository/repositories and accession number(s) can be found below: https://www.ncbi.nlm.nih.gov/, PRJNA722880.

## Ethics statement

The studies involving human participants were reviewed and approved by the institutional review board of the West China Hospital Stomatology of Sichuan University (Approval number: WCHSIRB-D-2018-101). The patients/participants provided their written informed consent to participate in this study.

## Author contributions

YH: Validation, methodology, data curation, writing—original draft, and writing— review and editing. ZZ: Methodology, data curation, and writing— original draft. XP: Methodology, data curation. PA: Methodology and data curation. QH: Methodology and data curation. BR: Supervision. ML: Supervision. HW: Supervision. XXZ: Formal analysis and supervision. XDZ: Conceptualization, methodology. YM: Conceptualization, methodology, formal analysis, writing—review and editing. LC: Conceptualization, methodology, writing— review and editing, and funding acquisition. All authors contributed to the article and approved the submitted version.

## Funding

This study was supported by the National Natural Science Foundation of China, (81870759, 82071106, LC), Innovative Research Team Program of Sichuan Province (LC), Research Funding from West China School/Hospital of Stomatology Sichuan University RCDWJS2021-19.

## Acknowledgments

Thanks to all the volunteers who participated in this experiment. Thanks to the doctors and technologists from the Department of Otolaryngology-Head and Neck Surgery of West China Hospital of Sichuan University for their professional support.

## Conflict of interest

The authors declare that the research was conducted in the absence of any commercial or financial relationships that could be construed as a potential conflict of interest.

## Publisher’s note

All claims expressed in this article are solely those of the authors and do not necessarily represent those of their affiliated organizations, or those of the publisher, the editors and the reviewers. Any product that may be evaluated in this article, or claim that may be made by its manufacturer, is not guaranteed or endorsed by the publisher.

## Supplementary material

Supplementary Table 1 Weight values order of characteristic variables in oral microbiome.

Supplementary Table 2 Weight values order of characteristic variables in nasopharyngeal microbiome.

Supplementary Table 3 The samples information of each group of subjects.

Supplementary Table 4 The Sequencing information statistics.

Supplementary Figure 1 The diversities of oral, nasal and nasopharyngeal microbiome. **(A)** Shannon index on OTU level of oral, nasal and nasopharyngeal microbiome. **(B)** Venn graph of oral, nasal and nasopharyngeal microbiome. **(C)** PCoA based on bray_curtis distance of oral, nasal and nasopharyngeal microbiome. **(D)** Community barplot analysis on genus level of oral, nasal and nasopharyngeal microbiome. KQ represents oral microbiome, BQ represents nasal microbiome, BY represents nasopharyngeal microbiome.

Supplementary Figure 2 Genus level analysis of species differences. **(A)** Differences in the dominant species composition of oral microbiome among cancer group, inflammation group and healthy counterparts. **(B)** Differences in the dominant species composition of nasal cavity microbiome among cancer group, inflammation group and healthy counterparts. **(C)** Differences in the dominant species composition of nasopharyngeal microbiome among cancer group, inflammation group and healthy counterparts. oral represents oral microbiome, nasal represents nasal microbiome, naso represents nasopharyngeal microbiome.

Supplementary Figure 3 The Rarefaction Curve based on Shannon indexes.

## References

[B1] Breiman L. (2001). Random forests. Mach. Learn. 45, 5–32. doi: 10.1023/A:1010933404324

[B2] Caruana R. Niculescu-Mizil A. (2006). An empirical comparison of supervised learning algorithms. Proc. 23rd. Int. Conf. Mach. Learn. C., 161–168. doi: 10.1145/1143844.1143865

[B3] Chan K. C. A. Woo J. K. S. King A. Zee B. C. Y. Lam W. K. J. Chan S. L. . (2017). Analysis of plasma Epstein-Barr virus DNA to screen for nasopharyngeal cancer. N. Engl. J. Med. 377, 513–522. doi: 10.1056/NEJMoa1701717 28792880

[B4] Chen Y. P. Chan A. T. C. Le Q. T. Blanchard P. Sun Y. Jun Ma M. (2019). Nasopharyngeal carcinoma. Lancet 394, 64–80. doi: 10.1016/S0140-6736(19)30956-0 31178151

[B5] Cole J. R. Chai B. Farris R. J. Wang Q. Kulam S. A. Mcgarrell D. M. . (2005). The ribosomal database project (RDP-II): sequences and tools for high-throughput rRNA analysis. Nucleic Acids Res. 33, D294–D296. doi: 10.1093/nar/gki038 15608200 PMC539992

[B6] Debelius J. W. Huang T. Cai Y. Ploner A. Barrett D. Zhou X. . (2020). Subspecies niche speacialization in the oral microbiome is associated with nasopharyngeal carcinoma risk. mSystems 5, e00065–e00020. doi: 10.1128/mSystems.00065-20 32636333 PMC7343305

[B7] De Boeck I. Wittouck S. Wuyts S. Oerlemans E. F. M. Van Den Broek M. F. L. Vandenheuvel D. . (2017). Comparing the healthy nose and nasopharynx microbiota reveals continuity as well as niche-specificity. Front. Microbiol. 29, 2372. doi: 10.3389/fmicb.2017.02372 PMC5712567 29238339

[B8] Dewhirst F. E. Chen T. Izard J. Paster B. J. Tanner A. C. Yu W. H. . (2010). The human oral microbiome. J. Bacteriol. 192, 5002–5017. doi: 10.1128/JB.00542-10 20656903 PMC2944498

[B9] Doupe P. Faghmous J. Basu S. (2019). Machine learning for health services researchers. Value. Health 22, 808–815. doi: 10.1016/j.jval.2019.02.012 31277828

[B10] Fatima N. Khan Mm Ia K. (2019). L-asparaginase produced from soil isolates of pseudomonas aeruginosa shows potent anti-cancer activity on HeLa cells. Saudi. J. Biol. Sci. 26, 1146–1153. doi: 10.1016/j.sjbs.2019.05.001 31516343 PMC6737276

[B11] Ferlay J. Colombet M. Soerjomataram I. Mathers C. Parkin D. M. Piñeros M. . (2019). Estimating the global cancer incidence and mortality in 2018: GLOBOCAN sources and methods. Int. J. Cancer 144, 1941–1953. doi: 10.1002/ijc.31937 30350310

[B12] Fishbein A. Hammock B. D. Serhan C. N. Panigrahy D. (2021). Carcinogenesis: Failure of resolution of inflammation? Pharmacol. Ther. 218, 107670. doi: 10.1016/j.pharmthera.2020.107670 32891711 PMC7470770

[B13] Flemer B. Warren Rd Barrett Mp Cisek K. Das A. Jeffery Ib . (2018). The oral microbiota in colorectal cancer is distinctive and predictive. Gut 67, 1454–1463. doi: 10.1136/gutjnl-2017-314814 28988196 PMC6204958

[B14] Frisan T. Nagy N. Chioureas D. Terol M. Grasso F. Mg M. (2018). A bacterial genotoxin causes virus reactivation and genomic instability in Epstein-Barr virus infected epithelial cells pointing to a role of co-infection in viral oncogenesis. Int. J. Cancer 144, 98–109. doi: 10.1002/ijc.31652 29978480 PMC6587852

[B15] Fyhrquist N. Ruokolainen L. Suomalainen A. Lehtimaki S. Veckman V. Vendelin J. . (2014). Acinetobacter species in the skin microbiota protect against allergic sensitization and inflammation. J. Allergy Clin. Immunol. 134, 1301–1309.e1311. doi: 10.1016/j.jaci.2014.07.059 25262465

[B16] Gao Y. Liu Jj Zhu Sy Yi X. (2014). The diagnostic accuracy of ultrasonography versus endoscopy for primary nasopharyngeal carcinoma. PloS One 9, e90412. doi: 10.1371/journal.pone.0090412 24594807 PMC3940890

[B17] Gerner C. Costigliola V. Golubnitschaja O. (2019). Multiomic patterns in body fluids: Technological challenge with a great potential to implement the advanced paradigm of 3p medicine. Mass. Spectrom. Rev. 39 (5-6), 442–451. doi: 10.1002/mas.21612 31737933

[B18] Hao Y. Tang C. Du Q. Zhou X. Peng X. L C. (2021). Comparative analysis of oral microbiome from zang and han populations living at different altitudes. Arch. Oral. Biol. 121, 104986. doi: 10.1016/j.archoralbio.2020.104986 33246246

[B19] Hu Y. J. Wang Q. Jiang Y. T. Ma R. Xia W. W. Tang Z. S. . (2013). Characterization of oral bacterial diversity of irradiated patients by high-throughput sequencing. Int. J. Oral. Sci. 5, 21–25. doi: 10.1038/ijos.2013.15 23538641 PMC3632764

[B20] Hyde E. R. Andrade F. Vaksman Z. Parthasarathy K. Jiang H. Parthasarathy D. K. . (2014). Metagenomic analysis of nitrate-reducing bacteria in the oral cavity: implications for nitric oxide homeostasis. PloS One 9, e88645. doi: 10.1371/journal.pone.0088645 24670812 PMC3966736

[B21] Ishwaran H. (2007). Variable importance in binary regression trees and forests. Electronic. J. Stat 1, 519–537. doi: 10.1214/07-EJS039

[B22] Kamran S. C. Riaz N. N L. (2015). Nasopharyngeal carcinoma. Surg. Oncol. Clin. N. Am. 24, 547–561. doi: 10.1016/j.soc.2015.03.008 25979399

[B23] King A. D. Vlantis A. C. Bhatia K. S. Zee B. C. Woo J. K. Tse G. M. . (2011). Primary nasopharyngeal carcinoma diagnostic accuracy of MR imaging versus that of endoscopyand endoscopic biopsy. Radiology 258, 531–537. doi: 10.1148/radiol.10101241 21131580

[B24] Lee A. W. M. Ng W. T. Chan J. Y. W. Corry J. Mäkitie A. Mendenhall W. M. . (2019). Management of locally recurrent nasopharyngeal carcinoma. Cancer Treat Rev. 79, 101890. doi: 10.1016/j.ctrv.2019.101890 31470314

[B25] Lee H. M. Okuda Ks González Fe Patel V. (2019). Current perspectives on nasopharyngeal carcinoma. Adv. Exp. Med. Biol. 1164, 11–34. doi: 10.1007/978-3-030-22254-3_2 31576537

[B26] Li T. Guo X. Ji M. Li F. Wang H. Cheng W. . (2018). Establishment and validation of a two-step screening scheme for improved performance of serological screening of nasopharyngeal carcinoma. Cancer Med. 7, 1458–1467. doi: 10.1002/cam4.1345 29479857 PMC5911604

[B27] Li Q. Hu Y. Zhou X. Liu S. Han Q. Cheng L. (2020). Role of oral bacteria in the development of oral squamous cell carcinoma. Cancers (Basel). 12, 2797–2814. doi: 10.3390/cancers12102797 33003438 PMC7600411

[B28] Lim Y. Fukuma N. Totsika M. Kenny L. Morrison M. Punyadeera C. (2018). The performance of an oral microbiome biomarker panel in predicting oral cavity and oropharyngeal cancers. Front. Cell Infect. Microbiol. 8, 267. doi: 10.3389/fcimb.2018.00267 30123780 PMC6085444

[B29] Liu Z. Chang E. T. Liu Q. Cai Y. Zhang Z. Chen G. . (2016). Oral hygiene and risk of nasopharyngeal carcinoma-a population-based case-control study in China. Cancer Epidemiol. Biomarkers Prev. 25, 1201–1207. doi: 10.1158/1055-9965.EPI-16-0149 27197279 PMC4970945

[B30] Li J. Zou X. Wu Y. L. Guo J. C. Yun J. P. Xu M. . (2014). A comparison between the sixth and seventh editions of the UICC/AJCC staging system for nasopharyngeal carcinoma in a Chinese cohort. PloS One 9, e116261. doi: 10.1371/journal.pone.0116261 25536307 PMC4275293

[B31] Luna P. N. Hasegawa K. Ajami N. J. Espinola J. A. Henke D. M. Petrosino J. F. . (2018). The association between anterior nares and nasopharyngeal microbiota in infants hospitalized for bronchiolitis. Microbiome 6, 2. doi: 10.1186/s40168-017-0385-0 29298732 PMC5751828

[B32] Murata M. (2018). Inflammation and cancer. Environ. Health Prev. Med. 23, 50. doi: 10.1186/s12199-018-0740-1 30340457 PMC6195709

[B33] Mwangi J. Yin Y. Wang G. Yang M. Li Y. Zhang Z. . (2019) The antimicrobial peptide ZY4 combats multidrug-resistant pseudomonas aeruginosa and acinetobacter baumannii infection. Proc. Natl. Acad. Sci. U. S. A. 116 (52), 26516–26522. doi: 10.1073/pnas.1909585117 31843919 PMC6936460

[B34] Nejman D. Livyatan I. Fuks G. Gavert N. Zwang Y. Geller Lt . (2020). The human tumor microbiome is composed of tumor type–specific intracellular bacteria. Science 368, 973–980. doi: 10.1126/science.aay9189 32467386 PMC7757858

[B35] Ngamchuea K. Chaisiwamongkhol K. Batchelor-Mcauley C. Compton R. G. (2017). Chemical analysis in saliva and the search for salivary biomarkers - a tutorial review. Analyst 143, 81–99. doi: 10.1039/C7AN01571B 29149225

[B36] Panda M. Rai A. K. Rahman T. Das A. Das R. Sarma A. . (2020). Alterations of salivary microbial community associated with oropharyngeal and hypopharyngeal squamous cell carcinoma patients. Arch. Microbiol. 202, 785–805. doi: 10.1007/s00203-019-01790-1 31832691

[B37] Quince C. Lanzen A. Curtis T. P. Davenport R. J. Hall N. Head I. M. . (2009). Accurate determination of microbial diversity from 454 pyrosequencing data. Nat. Methods 6, 639–641. doi: 10.1038/nmeth.1361 19668203

[B38] Ren Z. Li A. Jiang J. Zhou L. Yu Z. Lu H. . (2018). Gut microbiome analysis as a tool towards targeted non-invasive biomarkers for early hepatocellular carcinoma. Gut 68, 1014–1023. doi: 10.1136/gutjnl-2017-315084 30045880 PMC6580753

[B39] Sato Y. Kubo T. Morimoto K. Yanagihara K. Seyama T. (2016). High mannose-binding pseudomonas fluorescens lectin (PFL) downregulates cell surface integrin/EGFR and induces autophagy in gastric cancer cells. BMC Cancer 16, 63. doi: 10.1186/s12885-016-2099-2 26850110 PMC4744433

[B40] Sato Y. Matsubara K. Kubo T. Sunayama H. Hatori Y. Morimoto K. . (2019). High mannose binding lectin (PFL) from pseudomonas fluorescens down-regulates cancer-associated integrins and immune checkpoint ligand B7-H4. Cancers (Basel). 11, 604. doi: 10.3390/cancers11050604 31052260 PMC6562446

[B41] Seo G. Lee G. Kim M. J. Baek S.-H. Choi M. Ku K. B. . (2020). Rapid detection of COVID-19 causative virus (SARS-CoV-2) in human nasopharyngeal swab specimens using field-effect transistor-based biosensor. ACS Nano. 14, 5135–5142. doi: 10.1021/acsnano.0c02823 32293168

[B42] Song P. Wu L. Guan W. (2015). Dietary nitrates, nitrites, and nitrosamines intake and the risk of gastric cancer: A meta-analysis. Nutrients 7, 9872–9895. doi: 10.3390/nu7125505 26633477 PMC4690057

[B43] Sze M. A. Schloss P. D. (2018). Leveraging existing 16S rRNA gene surveys to identify reproducible biomarkers in individuals with colorectal tumors. MBio 9, e00630-18. doi: 10.1128/mBio.00630-18 29871916 PMC5989068

[B44] Tahmourespour A. Ahmadi A. M F. (2020). The anti-tumor activity of exopolysaccharides from pseudomonas strains against HT-29 colorectal cancer cell line. Int. J. Biol. Macromol. 149, 1072–1076. doi: 10.1016/j.ijbiomac.2020.01.268 32004609

[B45] Teng F. Yang F. Huang S. Bo C. Xu Z. Z. Amir A. . (2015). Prediction of early childhood caries *via* spatial-temporal variations of oral microbiota. Cell Host Microbe 18, 296–306. doi: 10.1016/j.chom.2015.08.005 26355216

[B46] Tsao S. W. Tsang C. M. Lo K. W. (2017). Epstein-Barr Virus infection and nasopharyngeal carcinoma. Philos. Trans. R. Soc. Lond. B. Biol. Sci. 372, 20160270–20160284. doi: 10.1098/rstb.2016.0270 28893937 PMC5597737

[B47] Turkoz F. P. (2011). Risk factors of nasopharyngeal carcinoma in Turkey - an epidemiological survey of the Anatolian society of medical oncology. Asian Pacific. J. Cancer Prev. 12, 3017. 22393983

[B48] Wang A. H. Li M. Li C. Q. Kou G. J. Zuo X. L. Li Y. Q. (2016). Human colorectal mucosal microbiota correlates with its host niche physiology revealed by endomicroscopy. Sci. Rep. 6, 21952. doi: 10.1038/srep21952 26916597 PMC4768150

[B49] Wolf P. Elsasser-Beile U. (2009). Pseudomonas exotoxin a: from virulence factor to anti-cancer agent. Int. J. Med. Microbiol. 299, 161–176. doi: 10.1016/j.ijmm.2008.08.003 18948059

[B50] Wu H. Ma Y. Peng X. Qiu W. Kong L. Ren B. . (2020). Antibiotic-induced dysbiosis of the rat oral and gut microbiota and resistance to salmonella. Arch. Oral. Biol. 114, 104730. doi: 10.1016/j.archoralbio.2020.104730 32371145

[B51] Xiao X. Zhang Z. Chang E. T. Liu Z. Liu Q. Cai Y. . (2018). Medical history, medication use, and risk of nasopharyngeal carcinoma. Am. J. Epidemiol. 187, 2117–2125. doi: 10.1093/aje/kwy095 29701753 PMC6166212

[B52] Xu Y. Teng F. Huang S. Lin Z. Yuan X. Zeng X. . (2014). Changes of saliva microbiota in nasopharyngeal carcinoma patients under chemoradiation therapy. Arch. Oral. Biol. 59, 176–186. doi: 10.1016/j.archoralbio.2013.10.011 24370189

[B53] Yang J. Mcdowell A. Kim E. K. Seo H. Lee W. H. Moon C. M. . (2019). Development of a colorectal cancer diagnostic model and dietary risk assessment through gut microbiome analysis. Exp. Mol. Med. 51, 1–15. doi: 10.1038/s12276-019-0313-4 PMC6802675 31582724

[B54] Yang J. Mu X. Wang Y. Zhu D. Zhang J. Liang C. . (2018). Dysbiosis of the salivary microbiome is associated with non-smoking female lung cancer and correlated with immunocytochemistry markers. Front. Oncol. 8, 520. doi: 10.3389/fonc.2018.00520 30524957 PMC6256243

[B55] Yin X. Gu X. Yin T. Wen H. Gao X. Zheng X. (2016). Study of enteropathogenic bacteria in children with acute diarrhoea aged from 7 to 10 years in xuzhou, China. Microb. Pathogen. 91, 41–45. doi: 10.1016/j.micpath.2015.11.027 26657723

[B56] Yu G. Phillips S. Gail M. H. Goedert Jj Humphrys M. S. Ravel J. . (2017). The effect of cigarette smoking on the oral and nasal microbiota. Microbiome 5, 3. doi: 10.1186/s40168-016-0226-6 28095925 PMC5240432

[B57] Zhang W. Guo Q. Liu G. Zheng F. Chen J. Huang D. . (2019). NKILA represses nasopharyngeal carcinoma carcinogenesis and metastasis by NF-κB pathway inhibition. PloS Genet. 15, e1008325. doi: 10.1371/journal.pgen.1008325 31430288 PMC6716677

[B58] Zhou X. Hao Y. Peng X. Li B. Han Q. Ren B. . (2021). The clinical potential of oral microbiota as a screening tool for oral squamous cell carcinomas. Front. Cell Infect. Microbiol. 11, 728933. doi: 10.3389/fcimb.2021.728933 34485181 PMC8416267

[B59] Zhu X. X. Yang X. J. Chao Y. L. Zheng H. M. Sheng H. F. Liu H. Y. . (2017). The potential effect of oral microbiota in the prediction of mucositis during radiotherapy for nasopharyngeal carcinoma. EBioMedicine 18, 23–31. doi: 10.1016/j.ebiom.2017.02.002 28216066 PMC5405060

[B60] Zhu C. Yuan C. Wei F. Q. Sun X. Y. Zheng S. G. (2020). Intraindividual variation and personal specificity of salivary microbiota. J. Dent. Res. 99, 1062–1071. doi: 10.1177/0022034520917155 32374655

