# Supplementary material for: The human oral – nasopharynx microbiome as a risk screening tool for nasopharyngeal carcinoma
Source: Front Cell Infect Microbiol. 2022 Nov 30;12:1013920. doi: 10.3389/fcimb.2022.1013920 (PMC9748088; doi:10.3389/fcimb.2022.1013920)
Supplement: Supplementary Table 1 — Weight values order of characteristic variables in oral microbiome. [file DataSheet_1.zip › Supplement material/Supplementary_Material.docx]

Supplementary Material

# Characteristics analysis of oral, nasal and nasopharyngeal microbiome

The alpha diversity index Shannon (**Figure S1A**) showed that the diversity of oral microbiome was the highest (*P*＜0.001). The beta diversity of PCoA analysis (**Figure S1B**) showed that significant differences existed among oral, nasal and nasopharyngeal microbiome (*P*＜0.05；R=0.9627). The Venn graph (**Figure S1C**) of species composition illustrated that there were 599 common OTUs in three groups, account for 25.72%-37.94% of the total microbiome.

The composition of oral, nasal and nasopharyngeal microbiome was demonstrated on genus level (**Figure S1D**). In oral cavity, 16 genera had an average relative abundance of more than 1%. In nasal cavity, 9 genera had an average relative abundance of more than 1%. In nasopharynx, 11 genera had an average relative abundance of more than 1%. The top five genera of relative abundance in oral cavity were *Streptococcus*, *Porphyromonas*, *Neisseria*, *Prevotella* and *Veillonella*. The top five genera of relative abundance in nasal cavity were *Corynebacterium*, *Staphylococcus*, *Cutibacterium*, *Moraxella* and *unclassified_u_norank_d_Bacteria*. The top five genera of relative abundance in nasopharynx were *Corynebacterium*, *Staphylococcus*, *Pseudppmonas*, *unclassified_u_norank_d_Bacteria* and *Streptococcus*.

# Analysis of species differences on genus level of three habitats from three groups

In the oral habitat, the healthy counterparts had the highest relative abundance of *Haemophilus and Alloprevotella* (*P＜0.01*，**Figure S2A**). The cancer and inflammation groups had higher *Actinomyces* and *Granulicatella* (*P＜0.05, P＜0.01*). There was no significant difference in the dominant species of nasal cavity microbiome among the cancer group, inflammation group and healthy group (**Figure S2B**). In the nasopharynx habitat, the healthy counterparts had the highest relative abundance of *Pseudomonas* and *Acinetobacter* (*P＜0.01*, **Figure S2C**). According to the analysis of species differences on genus level, the dominant species of inflammation group were more similar to cancer group than healthy group.

# The weights of characteristic variables of NPC risk screening model based on oral or nasopharyngeal microbiome

The weights of characteristic variables were calculated following the above-built models. Considering the oral microbial model, the most important genera in weight value were *Graulicatella*, *Prevotella*, *Rhodococcus*, *Haemophilus* (**Table S1**). *Granulicatella* ranked first and fourth, suggesting that this genus is a key biomarker of the oral microbiome. For the nasopharyngeal microbial model, *Candidatus solibacter*, *Pseudomonas*, *Bradyrhizobium*, and *Reyranella* were the most important genera, ranked by the front of weight value (**Table S2**). Genus *Pseudomonas* ranked the second, third, and seventh, suggesting it as a key biomarker of the nasopharyngeal microbiome.

# The information of samples and sequencing coverage and quality statistics

In the study, a total of 139 microbial samples were collected from 40 healthy people and 39 patients with nasopharyngeal biopsy, including 40 and 39 oral, eight and 27 nasal cavity, nine and 16 nasopharyngeal microbial samples. Following the pathological biopsy results, the nasopharyngeal biopsy group was divided into cancer and inflammation sub-groups (**Table S3**). The healthy and inflammation sub-groups constituted the non-cancer group. The NPC and inflammation sub-groups constituted the high-risk group. A risk screening tool for NPC was established by 16S rDNA sequencing and random forest.

Purified amplicons were pooled in equimolar and paired-end sequenced (2 × 300) on an Illumina MiSeq platform (Illumina, San Diego,USA) according to the standard protocols by Majorbio Bio-Pharm Technology Co. Ltd. (Shanghai, China). Raw fastq files were quality-filtered by Trimmomatic and merged by FLASH with the following criteria: (i) The reads were truncated at any site receiving an average quality score <20 over a 50 bp sliding window. (ii) Sequences whose overlap being longer than 10 bp were merged according to their overlap with mismatch no more than 2 bp. (iii)Sequences of each sample were separated according to barcodes (exactly matching) and Primers (allowing 2 nucleotide mismatching), and reads containing ambiguous bases were removed. Operational taxonomic units (OTUs) were clustered with 97% similarity cutoff using UPARSE（version 7.1 http://drive5.com/uparse/) with a novel ‘greedy’ algorithm that performs chimera filtering and OTU clustering simultaneously. The taxonomy of each 16S rRNA gene sequence was analyzed by RDP Classifier algorithm (http://rdp.cme.msu.edu/) against the Silva 16S rRNA database using confidence threshold of 70%. The sequencing information statistics of each sample was shown in **Table S4**. The average number of sequences was more than 40,000, and the Shannon curves tended to be flat in the rarefaction curve (**Figure S3**).


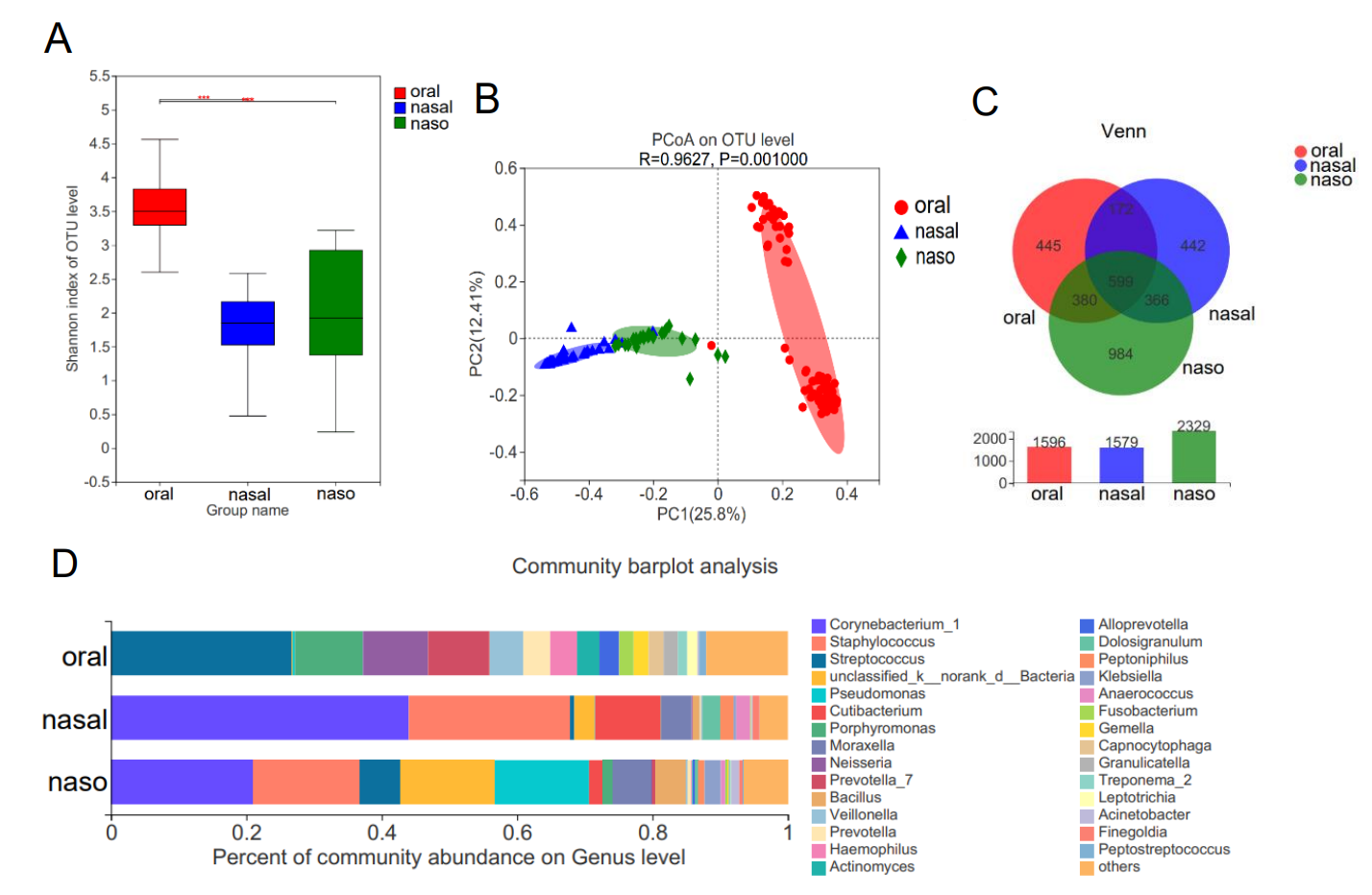


Figure S1. The diversities of oral, nasal and nasopharyngeal microbiome. (A) Shannon index on OTU level of oral, nasal and nasopharyngeal microbiome. (B) Venn graph of oral, nasal and nasopharyngeal microbiome. (C) PCoA based on bray_curtis distance of oral, nasal and nasopharyngeal microbiome. (D) Community barplot analysis on genus level of oral, nasal and nasopharyngeal microbiome. KQ represents oral microbiome, BQ represents nasal microbiome, BY represents nasopharyngeal microbiome.


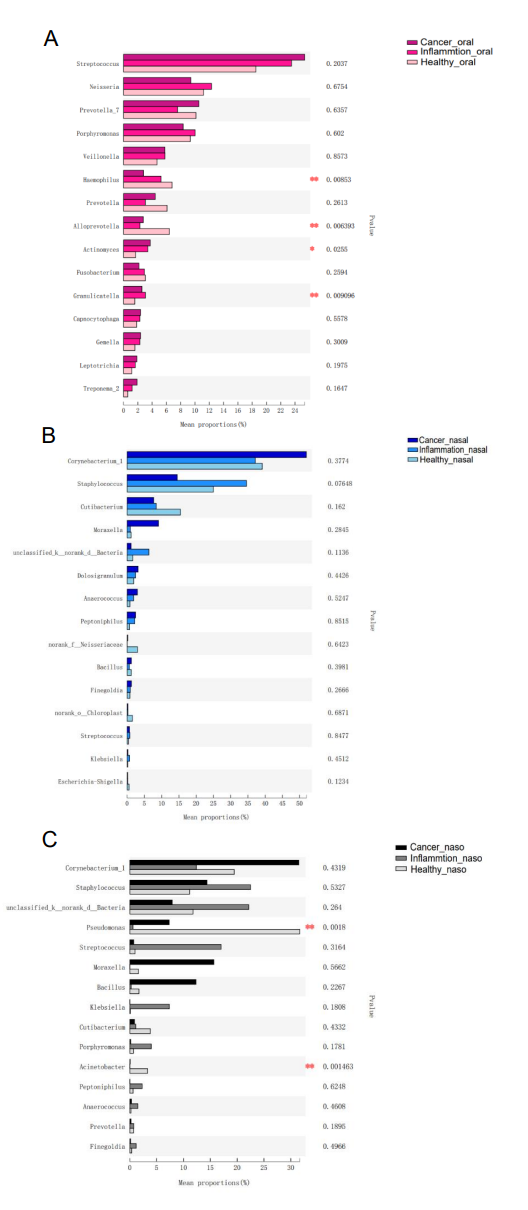


Figure S2. Genus level analysis of species differences. (A) Differences in the dominant species composition of oral microbiome among cancer group, inflammation group and healthy counterparts. (B) Differences in the dominant species composition of nasal cavity microbiome among cancer group, inflammation group and healthy counterparts. (C) Differences in the dominant species composition of nasopharyngeal microbiome among cancer group, inflammation group and healthy counterparts. oral represents oral microbiome, nasal represents nasal microbiome, naso represents nasopharyngeal microbiome.


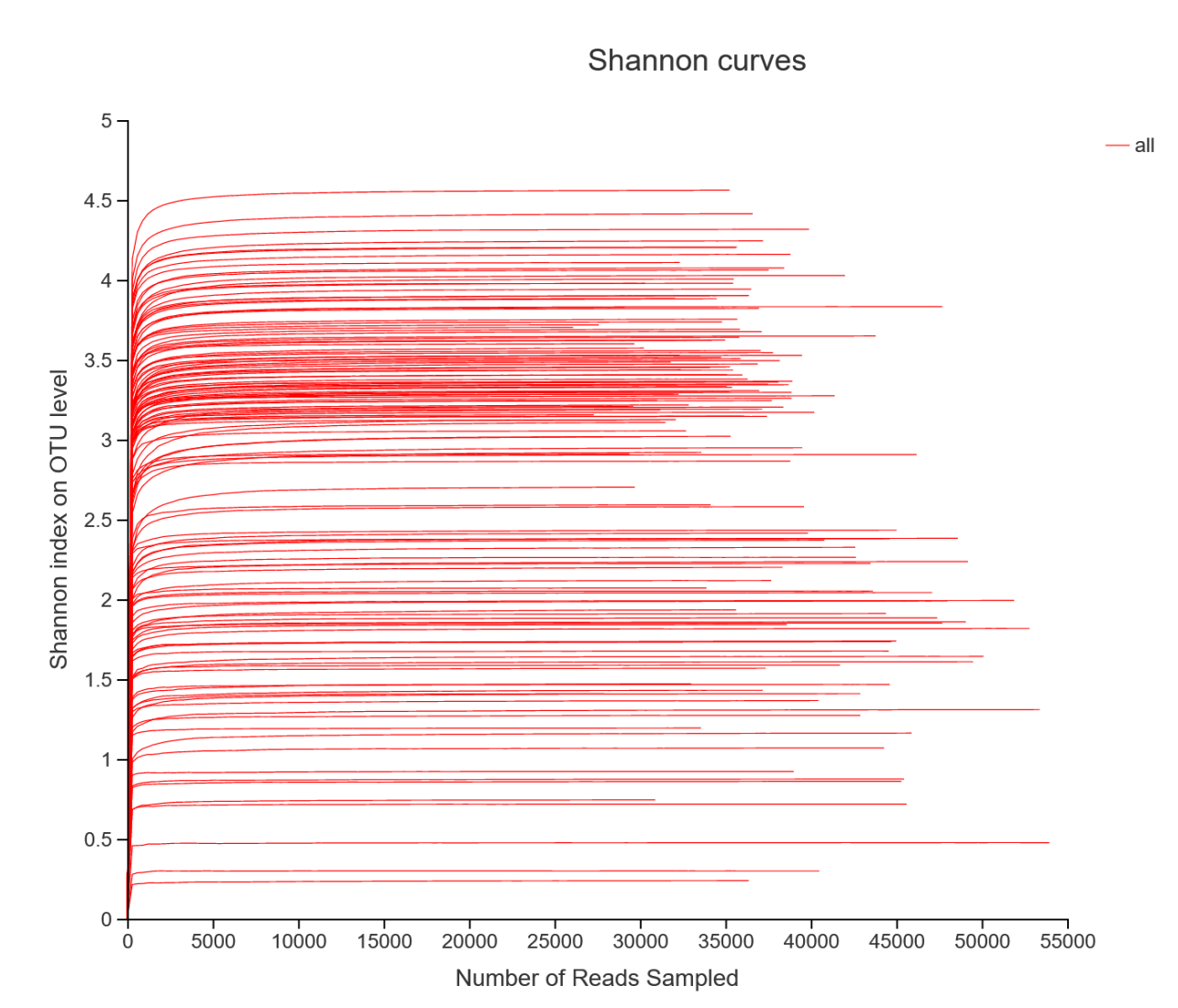


Figure S3. The Rarefaction Curve based on Shannon indexes.

Table S1. Weight values order of characteristic variables in oral microbiome.

| **Order** | **Weight Value** | **OTU** | **Genus** |
| --- | --- | --- | --- |
| 1 | 0.021668 | id |  |
| 2 | 0.006158 | OTU1134 | Granulicatella |
| 3 | 0.003995 | OTU3026 | unclassified_k__norank_d__Bacteria |
| 4 | 0.003575 | OTU3060 | Prevotella |
| 5 | 0.003053 | OTU465 | Granulicatella |
| 6 | 0.003002 | OTU3212 | Rhodococcus |
| 7 | 0.002611 | OTU2423 | Haemophilus |
| 8 | 0.002575 | OTU450 | unclassified_f__Neisseriaceae |
| 9 | 0.002382 | OTU211 | Olsenella |
| 10 | 0.002366 | OTU894 | Prevotella |
| 11 | 0.002355 | OTU383 | Streptococcus |
| 12 | 0.002216 | OTU895 | Streptococcus |
| 13 | 0.002098 | OTU111 | Streptococcus |
| 14 | 0.002077 | OTU472 | Dialister |
| 15 | 0.001992 | OTU2209 | Olsenella |

Table S2. Weight values order of characteristic variables in nasopharyngeal microbiome.

| **Order** | **Weight Value** | **OTU** | **Genus** |
| --- | --- | --- | --- |
| 1 | 0.003922 | id |  |
| 2 | 0.003842 | OTU1802 | Candidatus_Solibacter |
| 3 | 0.003837 | OTU900 | Pseudomonas |
| 4 | 0.003836 | OTU1497 | Pseudomonas |
| 5 | 0.00373 | OTU83 | Bradyrhizobium |
| 6 | 0.003593 | OTU1807 | Reyranella |
| 7 | 0.00355 | OTU2389 | norank_f__norank_o__norank_c__Subgroup_6 |
| 8 | 0.003463 | OTU1493 | Pseudomonas |
| 9 | 0.003451 | OTU1601 | norank_f__norank_o__norank_c__Alphaproteobacteria |
| 10 | 0.003437 | OTU2920 | Microbispora |
| 11 | 0.003352 | OTU1039 | Pseudomonas |
| 12 | 0.003349 | OTU1378 | Streptomyces |
| 13 | 0.003334 | OTU1909 | Aquicella |
| 14 | 0.003109 | OTU2317 | norank_f__norank_o__Acidobacteriales |
| 15 | 0.003067 | OTU2844 | Acidovorax |

Table S3. The samples information of each group of subjects.

| **Sample ID** | **Niche** | **Individual** | **Gender** | **Age** | **Pathologic_diagnosis** | **Group** |
| --- | --- | --- | --- | --- | --- | --- |
| BQ_01 | Nasal Cavity | 01 | F | 58 | Non-keratinic Carcinoma | NPC |
| BY_01 | Nasopharynx | 01 | F | 58 | Non-keratinic Carcinoma | NPC |
| KQ_01 | Oral Cavity | 01 | F | 58 | Non-keratinic Carcinoma | NPC |
| BY_06 | Nasopharynx | 06 | M | 53 | Non-keratinic Carcinoma | NPC |
| BQ_06 | Nasal Cavity | 06 | M | 53 | Non-keratinic Carcinoma | NPC |
| KQ_06 | Oral Cavity | 06 | M | 53 | Non-keratinic Carcinoma | NPC |
| KQ_12 | Oral Cavity | 12 | M | 57 | Non-keratinic Carcinoma | NPC |
| BY_14 | Nasopharynx | 14 | M | 45 | Non-keratinic Carcinoma | NPC |
| BQ_14 | Nasal Cavity | 14 | M | 45 | Non-keratinic Carcinoma | NPC |
| KQ_14 | Oral Cavity | 14 | M | 45 | Non-keratinic Carcinoma | NPC |
| BQ_19 | Nasal Cavity | 19 | F | 50 | Non-keratinic Carcinoma | NPC |
| KQ_19 | Oral Cavity | 19 | F | 50 | Non-keratinic Carcinoma | NPC |
| KQ_25 | Oral Cavity | 25 | M | 54 | Non-keratinic Carcinoma | NPC |
| BQ_29 | Nasal Cavity | 29 | M | 46 | Non-keratinic Carcinoma | NPC |
| KQ_29 | Oral Cavity | 29 | M | 46 | Non-keratinic Carcinoma | NPC |
| BQ_33 | Nasal Cavity | 33 | M | 70 | Non-keratinic Carcinoma | NPC |
| KQ_33 | Oral Cavity | 33 | M | 70 | Non-keratinic Carcinoma | NPC |
| KQ_35 | Oral Cavity | 35 | M | 20 | Non-keratinic Carcinoma | NPC |
| KQ_36 | Oral Cavity | 36 | M | 37 | Non-keratinic Carcinoma | NPC |
| BQ_36 | Nasal Cavity | 36 | M | 37 | Non-keratinic Carcinoma | NPC |
| BY_41 | Nasopharynx | 41 | F | 41 | Non-keratinic Carcinoma | NPC |
| BQ_41 | Nasal Cavity | 41 | F | 41 | Non-keratinic Carcinoma | NPC |
| KQ_41 | Oral Cavity | 41 | F | 41 | Non-keratinic Carcinoma | NPC |
| BQ_43 | Nasal Cavity | 43 | M | 35 | Non-keratinic Carcinoma | NPC |
| KQ_43 | Oral Cavity | 43 | M | 35 | Non-keratinic Carcinoma | NPC |
| BQ_45 | Nasal Cavity | 45 | F | 43 | Non-keratinic Carcinoma | NPC |
| KQ_45 | Oral Cavity | 45 | F | 43 | Non-keratinic Carcinoma | NPC |
| BQ_47 | Nasal Cavity | 47 | F | 50 | Non-keratinic Carcinoma | NPC |
| KQ_47 | Oral Cavity | 47 | F | 50 | Non-keratinic Carcinoma | NPC |
| BQ_48 | Nasal Cavity | 48 | F | 50 | Non-keratinic Carcinoma | NPC |
| BY_48 | Nasopharynx | 48 | F | 50 | Non-keratinic Carcinoma | NPC |
| KQ_48 | Oral Cavity | 48 | F | 50 | Non-keratinic Carcinoma | NPC |
| BQ_57 | Nasal Cavity | 57 | M | 29 | Non-keratinic Carcinoma | NPC |
| BY_57 | Nasopharynx | 57 | M | 29 | Non-keratinic Carcinoma | NPC |
| KQ_57 | Oral Cavity | 57 | M | 29 | Non-keratinic Carcinoma | NPC |
| BQ_59 | Nasal Cavity | 59 | M | 37 | Non-keratinic Carcinoma | NPC |
| BY_59 | Nasopharynx | 59 | M | 37 | Non-keratinic Carcinoma | NPC |
| KQ_59 | Oral Cavity | 59 | M | 37 | Non-keratinic Carcinoma | NPC |
| BQ_62 | Nasal Cavity | 62 | F | 53 | Non-keratinic Carcinoma | NPC |
| BY_62 | Nasopharynx | 62 | F | 53 | Non-keratinic Carcinoma | NPC |
| KQ_62 | Oral Cavity | 62 | F | 53 | Non-keratinic Carcinoma | NPC |
| S_KQ_01 | Oral Cavity | S_01 | F | 54 | Non-keratinic Carcinoma | NPC |
| S_KQ_02 | Oral Cavity | S_02 | M | 36 | Non-keratinic Carcinoma | NPC |
| S_KQ_04 | Oral Cavity | S_04 | M | 51 | Non-keratinic Carcinoma | NPC |
| S_KQ_05 | Oral Cavity | S_05 | M | 40 | Non-keratinic Carcinoma | NPC |
| S_KQ_06 | Oral Cavity | S_06 | F | 55 | Non-keratinic Carcinoma | NPC |
| S_KQ_08 | Oral Cavity | S_08 | F | 37 | Non-keratinic Carcinoma | NPC |
| S_KQ_09 | Oral Cavity | S_09 | M | 37 | Non-keratinic Carcinoma | NPC |
| S_KQ_10 | Oral Cavity | S_10 | M | 20 | Non-keratinic Carcinoma | NPC |
| S_KQ_11 | Oral Cavity | S_11 | M | 43 | Non-keratinic Carcinoma | NPC |
| BQ_03 | Nasal Cavity | 03 | F | 43 | Chronic mucosal inflammation with lymphoproliferative tissue | Inflamation |
| KQ_03 | Oral Cavity | 03 | F | 43 | Chronic mucosal inflammation with lymphoproliferative tissue | Inflamation |
| BQ_04 | Nasal Cavity | 04 | F | 51 | Chronic mucosal inflammation with lymphoproliferative tissue | Inflamation |
| BY_04 | Nasopharynx | 04 | F | 51 | Chronic mucosal inflammation with lymphoproliferative tissue | Inflamation |
| KQ_04 | Oral Cavity | 04 | F | 51 | Chronic mucosal inflammation with lymphoproliferative tissue | Inflamation |
| BQ_07 | Nasal Cavity | 07 | M | 20 | Chronic mucosal inflammation with lymphoproliferative tissue | Inflamation |
| KQ_07 | Oral Cavity | 07 | M | 20 | Chronic mucosal inflammation with lymphoproliferative tissue | Inflamation |
| BY_16 | Nasopharynx | 16 | F | 41 | Chronic mucosal inflammation with lymphoproliferative tissue | Inflamation |
| KQ_16 | Oral Cavity | 16 | F | 41 | Chronic mucosal inflammation with lymphoproliferative tissue | Inflamation |
| BQ_16 | Nasal Cavity | 16 | F | 41 | Chronic mucosal inflammation with lymphoproliferative tissue | Inflamation |
| BY_17 | Nasopharynx | 17 | M | 29 | Chronic mucosal inflammation with lymphoproliferative tissue | Inflamation |
| KQ_17 | Oral Cavity | 17 | M | 29 | Chronic mucosal inflammation with lymphoproliferative tissue | Inflamation |
| BQ_17 | Nasal Cavity | 17 | M | 29 | Chronic mucosal inflammation with lymphoproliferative tissue | Inflamation |
| BY_18 | Nasopharynx | 18 | F | 29 | Chronic mucosal inflammation with lymphoproliferative tissue | Inflamation |
| KQ_18 | Oral Cavity | 18 | F | 29 | Chronic mucosal inflammation with lymphoproliferative tissue | Inflamation |
| BQ_18 | Nasal Cavity | 18 | F | 29 | Chronic mucosal inflammation with lymphoproliferative tissue | Inflamation |
| BY_27 | Nasopharynx | 27 | F | 49 | Chronic mucosal inflammation with lymphoproliferative tissue | Inflamation |
| KQ_27 | Oral Cavity | 27 | F | 49 | Chronic mucosal inflammation with lymphoproliferative tissue | Inflamation |
| BQ_27 | Nasal Cavity | 27 | F | 49 | Chronic mucosal inflammation with lymphoproliferative tissue | Inflamation |
| KQ_30 | Oral Cavity | 30 | F | 50 | Chronic mucosal inflammation with lymphoproliferative tissue | Inflamation |
| BQ_30 | Nasal Cavity | 30 | F | 50 | Chronic mucosal inflammation with lymphoproliferative tissue | Inflamation |
| BQ_52 | Nasal Cavity | 52 | F | 28 | Chronic mucosal inflammation with lymphoproliferative tissue | Inflamation |
| KQ_52 | Oral Cavity | 52 | F | 28 | Chronic mucosal inflammation with lymphoproliferative tissue | Inflamation |
| BQ_55 | Nasal Cavity | 55 | F | 46 | Chronic mucosal inflammation with lymphoproliferative tissue | Inflamation |
| BY_55 | Nasopharynx | 55 | F | 46 | Chronic mucosal inflammation with lymphoproliferative tissue | Inflamation |
| KQ_55 | Oral Cavity | 55 | F | 46 | Chronic mucosal inflammation with lymphoproliferative tissue | Inflamation |
| BY_56 | Nasopharynx | 56 | F | 50 | Chronic mucosal inflammation with lymphoproliferative tissue | Inflamation |
| KQ_56 | Oral Cavity | 56 | F | 50 | Chronic mucosal inflammation with lymphoproliferative tissue | Inflamation |
| BQ_56 | Nasal Cavity | 56 | F | 50 | Chronic mucosal inflammation with lymphoproliferative tissue | Inflamation |
| BQ_68 | Nasal Cavity | 68 | F | 53 | Chronic mucosal inflammation with lymphoproliferative tissue | Inflamation |
| BY_68 | Nasopharynx | 68 | F | 53 | Chronic mucosal inflammation with lymphoproliferative tissue | Inflamation |
| KQ_68 | Oral Cavity | 68 | F | 53 | Chronic mucosal inflammation with lymphoproliferative tissue | Inflamation |
| CYY | Oral Cavity | CYY | F | 27 | / | Healthy |
| DonorA | Oral Cavity | DonorA | M | 30 | / | Healthy |
| HN | Oral Cavity | HN | F | 27 | / | Healthy |
| QY | Oral Cavity | QY | M | 24 | / | Healthy |
| SH_KQ_01 | Oral Cavity | SH_01 | M | 38 | / | Healthy |
| SH_KQ_02 | Oral Cavity | SH_02 | M | 65 | / | Healthy |
| SH_KQ_03 | Oral Cavity | SH_03 | F | 36 | / | Healthy |
| SH_KQ_04 | Oral Cavity | SH_04 | M | 66 | / | Healthy |
| SH_KQ_05 | Oral Cavity | SH_05 | F | 57 | / | Healthy |
| SH_KQ_06 | Oral Cavity | SH_06 | F | 60 | / | Healthy |
| SH_KQ_07 | Oral Cavity | SH_07 | F | 56 | / | Healthy |
| SH_KQ_08 | Oral Cavity | SH_08 | M | 29 | / | Healthy |
| SH_KQ_09 | Oral Cavity | SH_09 | M | 41 | / | Healthy |
| SH_KQ_10 | Oral Cavity | SH_10 | M | 28 | / | Healthy |
| SH_KQ_11 | Oral Cavity | SH_11 | F | 52 | / | Healthy |
| SH_KQ_12 | Oral Cavity | SH_12 | M | 47 | / | Healthy |
| SH_KQ_13 | Oral Cavity | SH_13 | F | 47 | / | Healthy |
| SH_KQ_14 | Oral Cavity | SH_14 | M | 55 | / | Healthy |
| SH_KQ_15 | Oral Cavity | SH_15 | M | 45 | / | Healthy |
| SH_KQ_16 | Oral Cavity | SH_16 | M | 46 | / | Healthy |
| SH_KQ_17 | Oral Cavity | SH_17 | M | 46 | / | Healthy |
| SH_KQ_18 | Oral Cavity | SH_18 | F | 46 | / | Healthy |
| SH_KQ_19 | Oral Cavity | SH_19 | F | 53 | / | Healthy |
| SH_KQ_20 | Oral Cavity | SH_20 | M | 26 | / | Healthy |
| WHLT | Oral Cavity | WHLT | F | 28 | / | Healthy |
| WHYT | Oral Cavity | WHYT | F | 29 | / | Healthy |
| WZ | Oral Cavity | WZ | M | 28 | / | Healthy |
| YJZ | Oral Cavity | YJZ | M | 27 | / | Healthy |
| ZCG | Oral Cavity | ZCG | M | 26 | / | Healthy |
| BY_60 | Nasopharynx | 60 | M | 25 | / | Healthy |
| BQ_60 | Nasal Cavity | 60 | M | 25 | / | Healthy |
| KQ_60 | Oral Cavity | 60 | M | 25 | / | Healthy |
| BY_63 | Nasopharynx | 63 | F | 32 | / | Healthy |
| KQ_63 | Oral Cavity | 63 | F | 32 | / | Healthy |
| BQ_63 | Nasal Cavity | 63 | F | 32 | / | Healthy |
| BQ_64 | Nasal Cavity | 64 | F | 28 | / | Healthy |
| BY_64 | Nasopharynx | 64 | F | 28 | / | Healthy |
| KQ_64 | Oral Cavity | 64 | F | 28 | / | Healthy |
| KQ_65 | Oral Cavity | 65 | M | 36 | / | Healthy |
| BY_66 | Nasopharynx | 66 | F | 54 | / | Healthy |
| KQ_66 | Oral Cavity | 66 | F | 54 | / | Healthy |
| BQ_66 | Nasal Cavity | 66 | F | 54 | / | Healthy |
| BY_67 | Nasopharynx | 67 | F | 57 | / | Healthy |
| BQ_67 | Nasal Cavity | 67 | F | 57 | / | Healthy |
| KQ_67 | Oral Cavity | 67 | F | 57 | / | Healthy |
| BY_69 | Nasopharynx | 69 | F | 61 | / | Healthy |
| KQ_69 | Oral Cavity | 69 | F | 61 | / | Healthy |
| BQ_69 | Nasal Cavity | 69 | F | 61 | / | Healthy |
| BY_70 | Nasopharynx | 70 | M | 34 | / | Healthy |
| KQ_70 | Oral Cavity | 70 | M | 34 | / | Healthy |
| BQ_70 | Nasal Cavity | 70 | M | 34 | / | Healthy |
| BQ_71 | Nasal Cavity | 71 | M | 26 | / | Healthy |
| BY_71 | Nasopharynx | 71 | M | 26 | / | Healthy |
| KQ_71 | Oral Cavity | 71 | M | 26 | / | Healthy |
| KQ_72 | Oral Cavity | 72 | M | 53 | / | Healthy |
| BY_73 | Nasopharynx | 73 | M | 29 | / | Healthy |
| KQ_73 | Oral Cavity | 73 | M | 29 | / | Healthy |

Table S4. The Sequencing information statistics.

| Sample\Info | Sequence_num | Base_num | Mean_length | Min_length | Max_length |
| --- | --- | --- | --- | --- | --- |
| BY_60 | 33831 | 13389771 | 395.7841 | 274 | 435 |
| KQ_45 | 41382 | 16288603 | 393.6157 | 277 | 412 |
| YJZ | 32642 | 12871645 | 394.3277 | 248 | 435 |
| KQ_36 | 40606 | 15970264 | 393.2981 | 276 | 434 |
| KQ_70 | 43936 | 17314589 | 394.0866 | 273 | 415 |
| KQ_18 | 42085 | 16596541 | 394.3576 | 277 | 405 |
| BY_59 | 36994 | 14209127 | 384.0927 | 264 | 459 |
| KQ_64 | 47549 | 18770906 | 394.7697 | 277 | 446 |
| KQ_27 | 41213 | 16266828 | 394.7014 | 277 | 440 |
| WHLT | 39469 | 15593828 | 395.0905 | 274 | 419 |
| BY_41 | 41645 | 15688610 | 376.7225 | 264 | 489 |
| DonorA | 58558 | 23082315 | 394.1787 | 345 | 443 |
| HN | 31880 | 12568110 | 394.2318 | 243 | 402 |
| BY_17 | 36410 | 14117619 | 387.7402 | 271 | 547 |
| KQ_68 | 48023 | 18945228 | 394.5032 | 276 | 516 |
| KQ_03 | 40586 | 16035650 | 395.103 | 277 | 436 |
| CYY | 35143 | 13844407 | 393.9449 | 231 | 400 |
| WZ | 30685 | 12076091 | 393.5503 | 263 | 412 |
| KQ_30 | 38929 | 15378796 | 395.0473 | 277 | 435 |
| KQ_17 | 46247 | 18252793 | 394.6806 | 276 | 442 |
| KQ_07 | 45093 | 17807628 | 394.9089 | 277 | 406 |
| KQ_43 | 44786 | 17662445 | 394.3742 | 277 | 547 |
| BY_66 | 37116 | 14269220 | 384.4493 | 264 | 455 |
| BY_55 | 38634 | 14734845 | 381.3958 | 261 | 520 |
| BY_67 | 32126 | 12387481 | 385.5905 | 272 | 459 |
| KQ_29 | 38469 | 15137596 | 393.5012 | 272 | 455 |
| KQ_12 | 57304 | 22606650 | 394.5039 | 277 | 417 |
| BY_73 | 42029 | 15943638 | 379.3485 | 260 | 464 |
| KQ_71 | 41161 | 16220872 | 394.0835 | 277 | 439 |
| KQ_52 | 46413 | 18236480 | 392.9175 | 264 | 409 |
| BY_57 | 34108 | 13460571 | 394.6456 | 271 | 523 |
| BY_16 | 51557 | 16642647 | 322.8009 | 264 | 522 |
| BY_14 | 30192 | 11514196 | 381.3658 | 261 | 459 |
| KQ_16 | 47708 | 18836503 | 394.829 | 248 | 410 |
| KQ_19 | 49046 | 19375671 | 395.051 | 277 | 417 |
| BY_01 | 41141 | 16218296 | 394.2125 | 264 | 442 |
| KQ_65 | 46111 | 18177100 | 394.2031 | 275 | 402 |
| KQ_04 | 45846 | 18070274 | 394.1516 | 273 | 528 |
| BY_62 | 35179 | 13469020 | 382.871 | 251 | 500 |
| KQ_56 | 37963 | 14982538 | 394.6616 | 272 | 459 |
| BY_56 | 47906 | 17953525 | 374.7657 | 260 | 525 |
| KQ_67 | 45534 | 17944589 | 394.0921 | 276 | 480 |
| KQ_55 | 45538 | 17948504 | 394.1434 | 277 | 414 |
| KQ_47 | 37312 | 14759520 | 395.5703 | 277 | 547 |
| KQ_57 | 40944 | 16166219 | 394.8373 | 276 | 401 |
| BY_06 | 31912 | 12706345 | 398.1682 | 265 | 434 |
| KQ_73 | 45737 | 18023326 | 394.0645 | 277 | 481 |
| KQ_33 | 44459 | 17506163 | 393.7597 | 272 | 401 |
| KQ_35 | 46060 | 18165752 | 394.3932 | 277 | 434 |
| BY_18 | 46036 | 16207687 | 352.0655 | 264 | 443 |
| BY_63 | 45850 | 17887983 | 390.1414 | 258 | 467 |
| BY_04 | 36896 | 14597557 | 395.6406 | 274 | 443 |
| BY_48 | 35855 | 14175106 | 395.3453 | 272 | 414 |
| KQ_62 | 48898 | 19271592 | 394.1182 | 277 | 411 |
| KQ_69 | 44292 | 17443842 | 393.8373 | 277 | 426 |
| BY_64 | 34902 | 13270225 | 380.2139 | 257 | 456 |
| KQ_48 | 37925 | 14970332 | 394.7352 | 272 | 463 |
| KQ_60 | 46151 | 18178087 | 393.8828 | 277 | 459 |
| ZCG | 34565 | 13632197 | 394.3931 | 263 | 401 |
| BY_69 | 36807 | 14556023 | 395.4689 | 273 | 455 |
| WHYT | 37370 | 14777760 | 395.4445 | 274 | 410 |
| KQ_06 | 32202 | 12724794 | 395.1554 | 272 | 450 |
| BY_71 | 37526 | 13059345 | 348.0079 | 220 | 519 |
| QY | 33352 | 13132916 | 393.767 | 386 | 400 |
| BY_70 | 33315 | 13176780 | 395.5209 | 274 | 417 |
| KQ_01 | 42229 | 16650510 | 394.2909 | 277 | 412 |
| KQ_14 | 40992 | 16154782 | 394.096 | 277 | 516 |
| KQ_66 | 40935 | 16129193 | 394.0196 | 264 | 413 |
| KQ_41 | 42751 | 16877690 | 394.7905 | 274 | 520 |
| KQ_25 | 41345 | 16329941 | 394.9677 | 274 | 435 |
| BY_27 | 45756 | 16614691 | 363.115 | 264 | 461 |
| BY_68 | 39062 | 15053969 | 385.3865 | 261 | 455 |
| KQ_63 | 44902 | 17745721 | 395.21 | 277 | 409 |
| KQ_59 | 46149 | 18165849 | 393.6347 | 277 | 409 |
| KQ_72 | 39973 | 15741490 | 393.8031 | 276 | 453 |
| SH_KQ_15 | 45353 | 16985646 | 374.5209 | 244 | 398 |
| S_KQ_11 | 40725 | 15285338 | 375.3306 | 244 | 386 |
| S_KQ_04 | 41998 | 15760065 | 375.2575 | 244 | 386 |
| SH_KQ_13 | 44298 | 16593387 | 374.5855 | 256 | 386 |
| S_KQ_10 | 44544 | 16700772 | 374.9275 | 314 | 386 |
| SH_KQ_19 | 48955 | 18367355 | 375.1885 | 257 | 392 |
| S_KQ_05 | 47385 | 17786116 | 375.3533 | 257 | 386 |
| SH_KQ_03 | 37430 | 14042370 | 375.1635 | 257 | 381 |
| SH_KQ_01 | 45710 | 17110651 | 374.3306 | 318 | 386 |
| SH_KQ_14 | 51094 | 19122691 | 374.2649 | 206 | 526 |
| SH_KQ_07 | 41313 | 15494933 | 375.0619 | 257 | 521 |
| SH_KQ_16 | 46525 | 17405747 | 374.116 | 290 | 525 |
| S_KQ_08 | 50429 | 18914937 | 375.0805 | 337 | 416 |
| S_KQ_06 | 45241 | 16960130 | 374.8841 | 257 | 386 |
| SH_KQ_12 | 46605 | 17459824 | 374.6341 | 257 | 386 |
| SH_KQ_17 | 46730 | 17490884 | 374.2967 | 206 | 386 |
| SH_KQ_20 | 50929 | 19024831 | 373.556 | 256 | 383 |
| SH_KQ_04 | 38813 | 14540565 | 374.6313 | 257 | 386 |
| S_KQ_02 | 39973 | 14980496 | 374.7654 | 257 | 386 |
| SH_KQ_05 | 35117 | 13184854 | 375.455 | 244 | 401 |
| S_KQ_01 | 41670 | 15614171 | 374.7101 | 258 | 408 |
| SH_KQ_10 | 39146 | 14654235 | 374.3482 | 206 | 383 |
| S_KQ_09 | 48491 | 18162320 | 374.5503 | 258 | 385 |
| SH_KQ_06 | 51678 | 19354954 | 374.5299 | 244 | 386 |
| SH_KQ_02 | 41894 | 15736950 | 375.6373 | 257 | 501 |
| SH_KQ_18 | 40232 | 15080062 | 374.8276 | 256 | 385 |
| SH_KQ_09 | 40930 | 15366684 | 375.4382 | 325 | 386 |
| SH_KQ_11 | 44633 | 16747960 | 375.2372 | 256 | 391 |
| SH_KQ_08 | 40758 | 15276497 | 374.8098 | 257 | 385 |
| BQ_56 | 46181 | 17284652 | 374.2806 | 233 | 517 |
| BQ_43 | 38755 | 14650331 | 378.0243 | 256 | 524 |
| BQ_60 | 46276 | 17215894 | 372.0264 | 244 | 517 |
| BQ_19 | 38100 | 14321342 | 375.8882 | 255 | 431 |
| BQ_62 | 55524 | 20542506 | 369.9753 | 205 | 446 |
| BQ_63 | 45074 | 16996701 | 377.0844 | 238 | 448 |
| BQ_64 | 41442 | 15616115 | 376.8186 | 244 | 483 |
| BQ_66 | 39941 | 15041373 | 376.5898 | 243 | 505 |
| BQ_67 | 53586 | 20095674 | 375.0172 | 244 | 400 |
| BQ_68 | 49620 | 18692392 | 376.7108 | 243 | 514 |
| BQ_69 | 47259 | 17842237 | 377.5416 | 224 | 454 |
| BQ_27 | 40087 | 14955174 | 373.0679 | 244 | 448 |
| BQ_48 | 51152 | 19297579 | 377.2595 | 244 | 472 |
| BQ_47 | 39677 | 14952662 | 376.8597 | 252 | 513 |
| BQ_45 | 43936 | 16570123 | 377.1423 | 206 | 482 |
| BQ_29 | 42276 | 15948008 | 377.2355 | 255 | 498 |
| BQ_41 | 54106 | 20353480 | 376.1779 | 244 | 381 |
| BQ_03 | 46517 | 17507252 | 376.3624 | 252 | 493 |
| BQ_04 | 54276 | 20343779 | 374.8209 | 243 | 380 |
| BQ_01 | 50864 | 18951010 | 372.582 | 244 | 424 |
| BQ_06 | 44149 | 16686671 | 377.9626 | 238 | 517 |
| BQ_07 | 48689 | 16017206 | 328.9697 | 204 | 544 |
| BQ_71 | 45945 | 17174593 | 373.8077 | 231 | 524 |
| BQ_70 | 43034 | 16185043 | 376.099 | 210 | 453 |
| BQ_59 | 50998 | 19120172 | 374.92 | 241 | 435 |
| BQ_14 | 47232 | 17740343 | 375.6001 | 226 | 537 |
| BQ_17 | 46119 | 17419679 | 377.7116 | 244 | 382 |
| BQ_16 | 47805 | 17958519 | 375.6619 | 244 | 441 |
| BQ_33 | 46037 | 17316671 | 376.1468 | 231 | 446 |
| BQ_18 | 39296 | 14765423 | 375.7488 | 206 | 386 |
| BQ_57 | 50052 | 18898129 | 377.5699 | 225 | 434 |
| BQ_30 | 45057 | 16931606 | 375.7819 | 238 | 424 |
| BQ_36 | 46821 | 17571278 | 375.2863 | 241 | 473 |
| BQ_52 | 48457 | 17797871 | 367.2921 | 233 | 506 |
| BQ_55 | 45130 | 16512734 | 365.8926 | 241 | 458 |
