# Supplementary figures and images for: The human oral – nasopharynx microbiome as a risk screening tool for nasopharyngeal carcinoma
Source: Front Cell Infect Microbiol. 2022 Nov 30;12:1013920. doi: 10.3389/fcimb.2022.1013920 (PMC9748088; doi:10.3389/fcimb.2022.1013920)

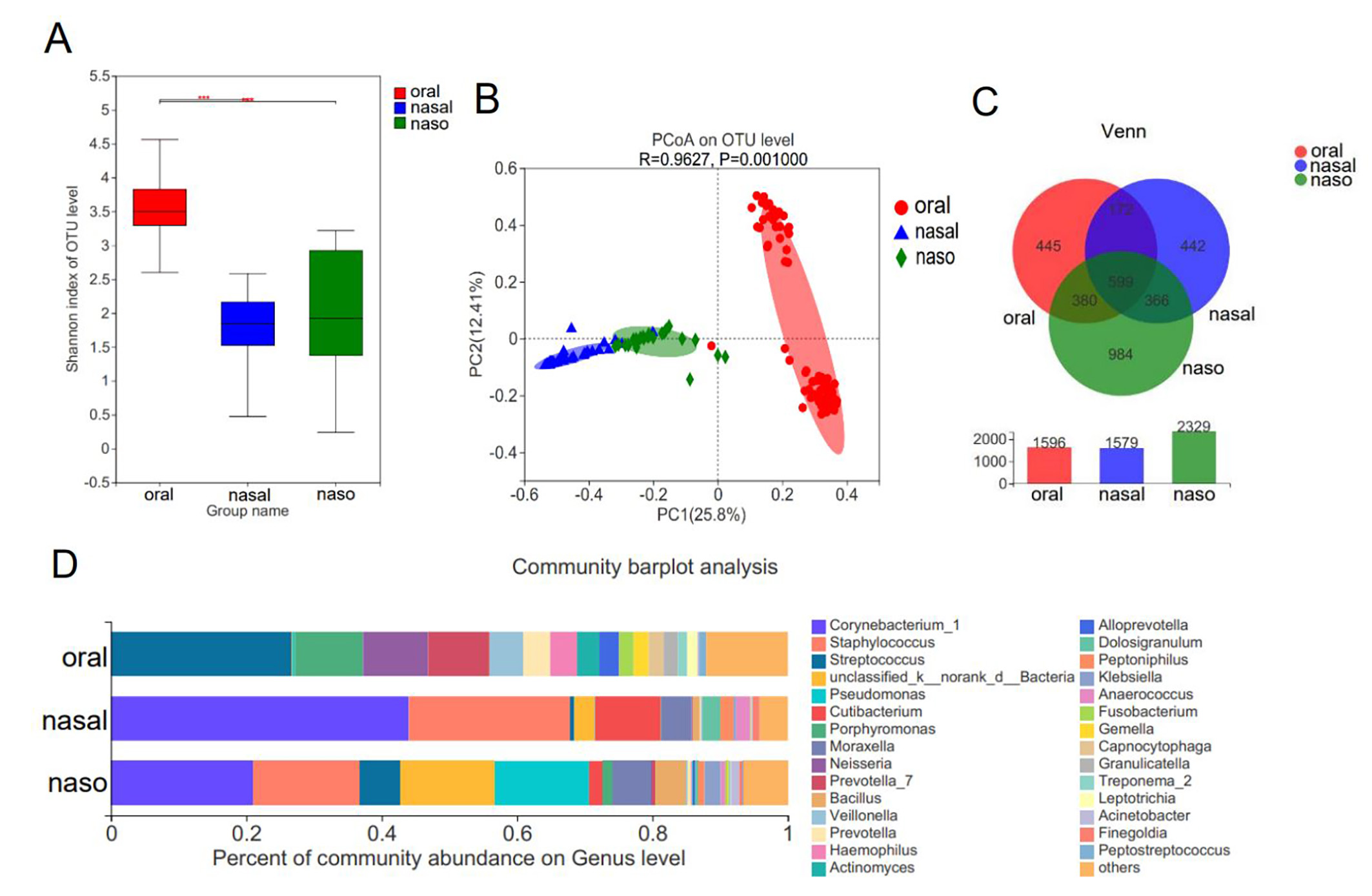

Supplement: Supplementary Table 1 — Weight values order of characteristic variables in oral microbiome. [file DataSheet_1.zip › Supplement material/Fig S1.JPG]

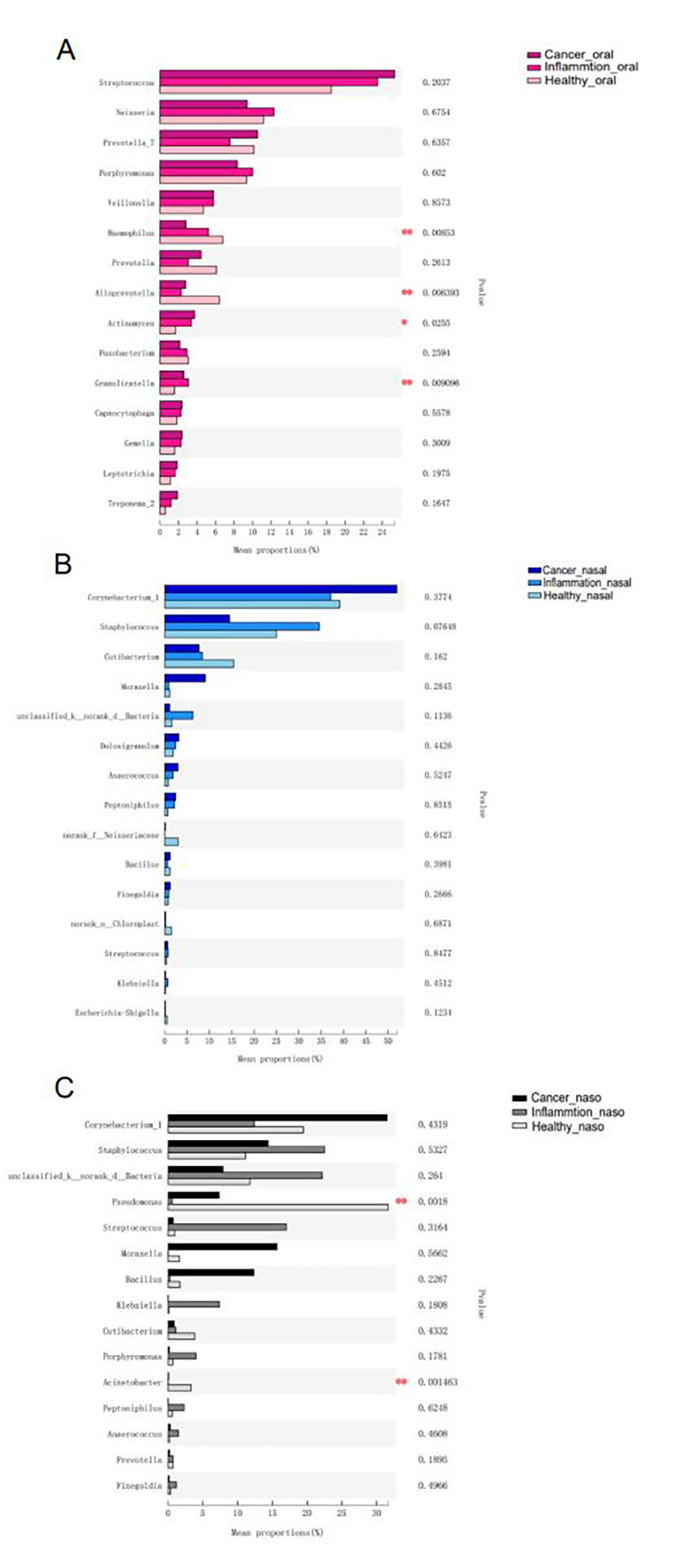

Supplement: Supplementary Table 1 — Weight values order of characteristic variables in oral microbiome. [file DataSheet_1.zip › Supplement material/Fig S2.JPG]

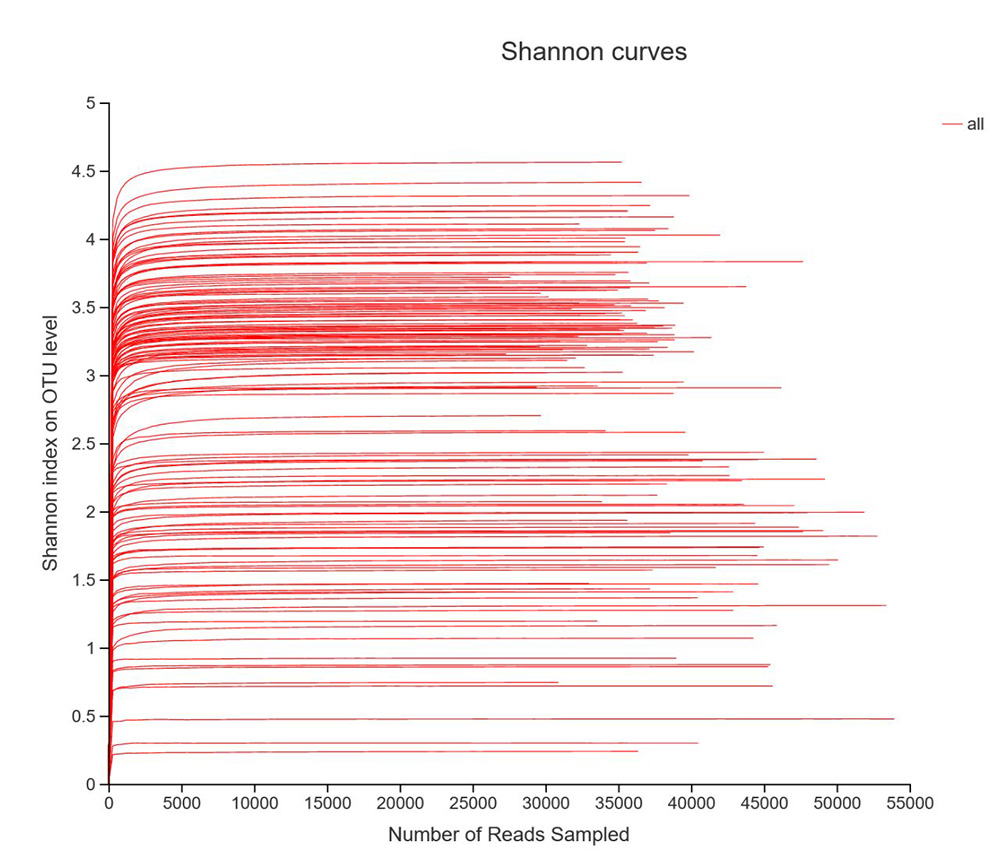

Supplement: Supplementary Table 1 — Weight values order of characteristic variables in oral microbiome. [file DataSheet_1.zip › Supplement material/Fig S3.JPG]
